# Base-pairing of uracil and 2,6-diaminopurine: from cocrystals to photoreactivity

**DOI:** 10.1016/j.isci.2024.109894

**Published:** 2024-05-07

**Authors:** Tomislav Stolar, Ben K.D. Pearce, Martin Etter, Khai-Nghi Truong, Tea Ostojić, Andraž Krajnc, Gregor Mali, Barbara Rossi, Krešimir Molčanov, Ivor Lončarić, Ernest Meštrović, Krunoslav Užarević, Luca Grisanti

**Affiliations:** 1 Ruđer Bošković Institute, Bijenička c. 54, 10000 Zagreb, Croatia; 2 Federal Institute for Materials Research and Testing (BAM), Richard-Willstätter-Straße 11, 12489 Berlin, Germany; 3 Johns Hopkins University, 3400 N. Charles Street, Baltimore, MD 21218, USA; 4 Deutsches Elektronen-Synchrotron (DESY), Notkestraße 85, 22607 Hamburg, Germany; 5 Rigaku Europe SE, Hugenottenallee 167, 63263 Neu-Isenburg, Germany; 6 National Institute of Chemistry, Hajdrihova 19, 1000 Ljubljana, Slovenia; 7 Elettra Sincrotrone Trieste, Strada Statale 14 km 163.5, 34149 Trieste, Italy; 8 Faculty of Chemical Engineering and Technology, University of Zagreb, Trg Marka Marulića 19, 10000 Zagreb, Croatia; 9 National Research Council - Materials Foundry Institute (CNR-IOM) c/o SISSA (International School for Advanced Studies), Via Bonomea 265, 34136 Trieste, Italy

**Keywords:** Chemistry, Theoretical photochemistry

## Abstract

We show that the non-canonical nucleobase 2,6-diaminopurine (D) spontaneously base pairs with uracil (U) in water and the solid state without the need to be attached to the ribose-phosphate backbone. Depending on the reaction conditions, D and U assemble in thermodynamically stable hydrated and anhydrated D-U base-paired cocrystals. Under UV irradiation, an aqueous solution of D-U base-pair undergoes photochemical degradation, while a pure aqueous solution of U does not. Our simulations suggest that D may trigger the U photodimerization and show that complementary base-pairing modifies the photochemical properties of nucleobases, which might have implications for prebiotic chemistry.

## Highlights

• Base-pairing in reaction conditions of different water availability and temperatures
• Crystal structures solved from electron and single-crystal X-ray diffraction data
• Photodegradation of base-pair under UV irradiation

## Graphical abstract

**Figure:**
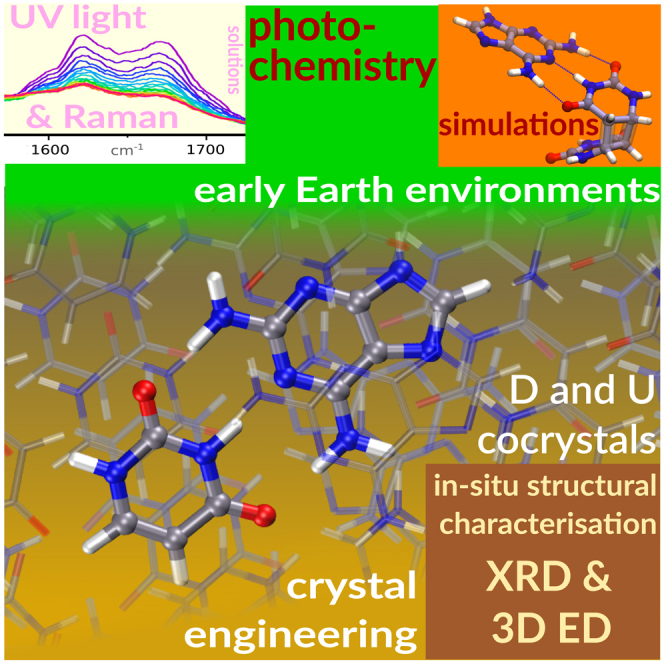

## Introduction

Canonical nucleobases are attached to the sugar-phosphate backbone in nucleic acids and act as molecular recognition units. Their complementary base-pairing is central to the transfer of genetic information.^1^ Moreover, base-pairing of free canonical nucleobases (without being attached to the sugar-phosphate backbone) might have been a starting point for the origin of nucleic acids.^2^ However, it has been known for almost 60 years that there is no molecular recognition and complementary base-pairing of free canonical nucleobases in water.^3^^,^^4^ Instead, they form energetically favored stacking structures and hydrogen bonds with water molecules.^5^ We recently showed that base-pairing of free canonical nucleobases does not occur even in the solid-state reaction environment devoid of water.^6^ Although it is unclear how the chemical selection of nucleobases occurred from a larger pool of similar molecules likely available on the prebiotic Earth,^7^^,^^8^^,^^9^^,^^10^^,^^11^ the formation of thermodynamically and kinetically stable base pairs on a monomer level might affect their resulting photochemical properties. Furthermore, the evidence for base-pairing of free canonical or non-canonical nucleobases in the solid state may be a piece of the puzzle, and the corresponding structural motifs might guide computational studies. The latter remains largely unexplored in the literature and serves as a motivation for this work.

**Figure 1:**
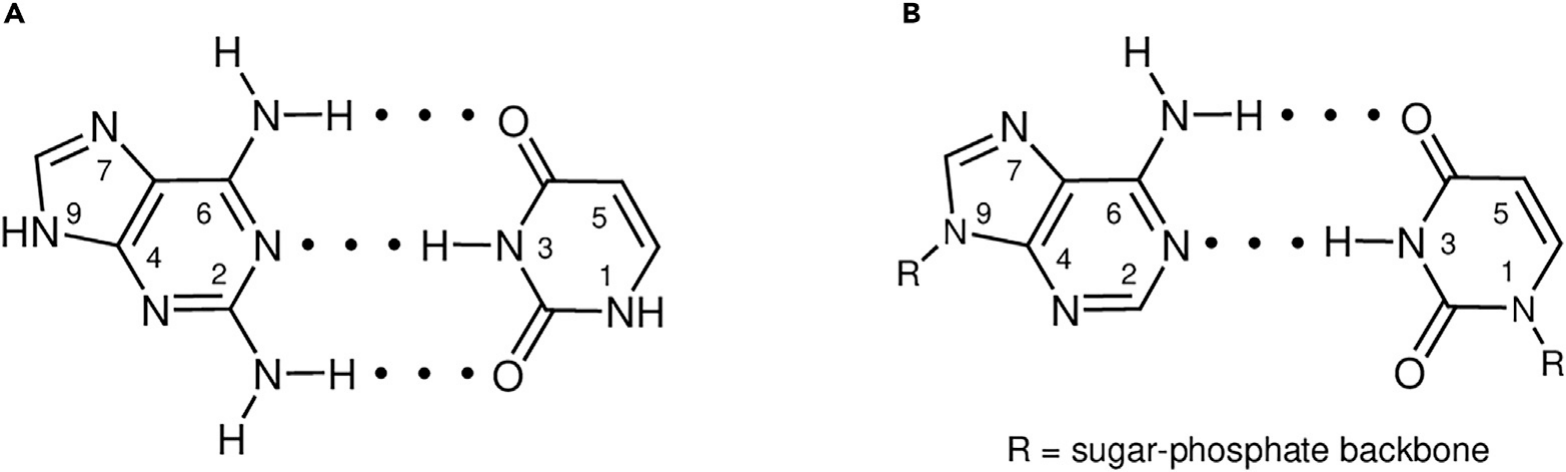
Molecular structures of investigated nucleobases (A) Molecular structures of D (left) and U (right) with highlighted hydrogen bonding complementarity. This base pair has the potential to form up to three intermolecular hydrogen bonds. (B) Molecular structures of A (left) and U (right) with Watson-Crick base-pairing motif by two intermolecular hydrogen bonds. This base-pairing occurs in nucleic acids but does not occur on the level of free nucleobases in the absence of a sugar-phosphate backbone.

Non-canonical nucleobases are intriguing, as they would have been present during chemical evolution, and some even play roles in life processes today. For example, D is present in meteorites alongside U, and its extraterrestrial origin is confirmed.^12^^,^^13^ When incorporated into DNA, D repairs UV-induced lesions.^14^ Nucleoside and nucleotide analogs of D are also products of selective prebiotic syntheses.^15^^,^^16^ Remarkably, D is even present in contemporary biology as it completely substitutes A in the genomes of a wide array of siphoviruses.^17^^,^^18^^,^^19^^,^^20^^,^^21^ Looking at the molecular level, D contains an additional amino group in the 2 position compared to A and can form up to three intermolecular hydrogen bonds with U (Figures 1A and 1B). On the other hand, Watson-Crick hydrogen bonding between A and U by two intermolecular hydrogen bonds occurs when attached to the sugar-phosphate backbone but is absent on the level of free nucleobases. If available under plausibly prebiotic conditions, thermodynamically more favorable D-U base-pairing is important in understanding the chemical evolution of these two nucleobases. For example, the complementary supramolecular assembly of D and U could affect their photostability under UV light.^22^ Importantly, solar radiation was likely the primary source of energy available on early Earth and a key factor for chemical reactivity and selection.^23^

In this work, we combine several state-of-the-art experimental and computational techniques to investigate the interaction of D and U, the formation of their assemblies in different states (solid, liquid, and in vacuum), and discuss the impact on photochemistry. We reveal the self-assembly of D-U cocrystal hydrate (D-U hyd) and anhydrate (D-U anhyd) under reaction conditions of different water availability (bulk water, stoichiometric water, water vapor, and dry state) and temperatures (low and elevated). We monitor the formation of D-U hyd and its wet/dry cycling to D-U anhyd by *in situ* synchrotron powder X-ray diffraction (PXRD). We elucidate the crystal structure of D-U hyd from single crystal X-ray diffraction (SCXRD) data and D-U anhyd from 3D electron diffraction (3D ED) data to study the crystal packing. We estimate favorable formation and stabilization energies of D-U base-paired cocrystals by periodic density functional theory (DFT) computation and reveal the formation of Watson-Crick dimers as a driving force for their assembly. To study the photostability of D-U base-paired cocrystal, we measure its aqueous solution under UV light by *in situ* UV resonance Raman (UVRR) spectroscopy. Our results show that the addition of D into an aqueous solution of U photochemically destabilizes U compared to its pure solutions. That is an important finding since U—and other canonical nucleobases in their free forms are photochemically stable when irradiated by UV light.^22^ On the other hand, well studied photoreactive pathways^24^^,^^25^ appear to be strongly dependent on the environment, and as we show in this work, the situation differs when non-covalent interactions between complementary nucleobases, such as D and U, are in place.^26^ Furthermore, we perform quantum-chemical and DFT simulations to show that D-U hydrogen bonding may promote photoreaction to form cyclobutane pyrimidine dimers (CPDs) of U. Thus, serving as a possible pathway for the main photochemical degradation channel of U that needs at least two U nucleobases to be in proximity to be effective.

## Results

**Figure 2:**
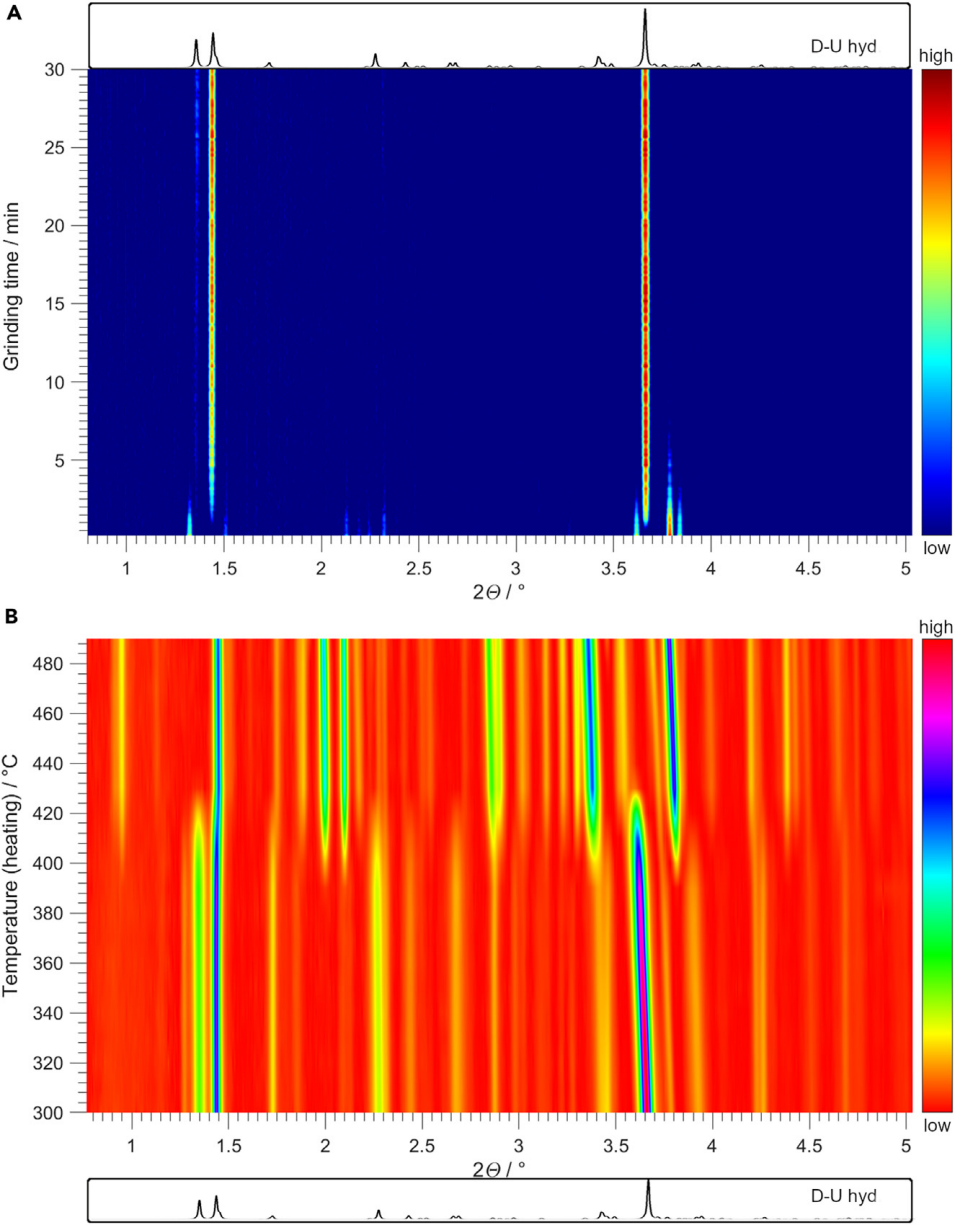
*In situ* PXRD monitoring of D-U cocrystals (A) *In situ* monitoring of grinding 200 mg of an equimolar amount of D and U with 20 μ L of H_2_O by synchrotron PXRD (λ = 0.20741 Å). A simulated PXRD pattern of D-U hyd is given in a box at the top. Intensity color code is given on the right. (B) *In situ* monitoring of heating D-U hyd in a sealed glass capillary by synchrotron PXRD (λ = 0.20741 Å). A simulated PXRD pattern of D-U hyd is given in a box at the bottom. Intensity color code is given on the right. Note: some Bragg reflections are shifting toward lower 2*Θ* values during heating due to the expansion of the unit cell.

Stirring an aqueous suspension of D and U for six days at room temperature resulted in a product primarily composed of D-U cocrystal hydrate (D-U hyd, Figures S1 and S2). Dry grinding of D and U for 2 h resulted in a product with an amorphous PXRD pattern (Figure S1) which, upon aging^27^ in H_2_O vapor for seven days at room temperature, transformed to D-U hyd (Figure S1). The input of mechanical energy, considered an important energy source for prebiotic chemistry,^28^^,^^29^^,^^30^^,^^31^^,^^32^ is not a prerequisite for D-U hyd formation in moisture. Gentle homogenization of D and U with the spatula and aging in H_2_O vapor at room temperature also resulted in the formation of D-U hyd (Figures S1 and S3). We monitored the grinding of D and U with a stoichiometric amount of H_2_O by *in situ* synchrotron PXRD (Figure 2A). It showed that D-U hyd started to form within the first minute of grinding, and the reaction mixture was phase pure by 30 min (Figures S4 and S5). Additionally, we studied the base-pairing of D and U under low-temperature conditions. After 30 min of grinding D and U with a stoichiometric amount of H_2_O at ca. 80 K, PXRD analysis confirmed the formation of D-U hyd (Figure S1).

We also performed wet-dry cycling^33^ in a sealed glass capillary and monitored it by *in situ* synchrotron PXRD (Figure 2B). D-U hyd was thermally stable until 410 K, when it started transforming into different crystalline phases (Figure S6). Rietveld analysis showed that after heating to 490 K (Figure S7), the solid sample was composed of D-U anhyd, D hydrate,^34^ and D anhydrate.^35^ Similarly, heating D-U hyd to 530 K in an open container (Figure S8) resulted in a solid sample composed of D-U hyd, D-U anhyd, D hydrate, and D anhydrate (Figure S9). The latter was consistent with the ^1^H-C^13^ cross-polarization magic-angle spinning nuclear magnetic resonance spectra which showed that the sample was not phase pure (Figure S10).

We also tested if D-U base-pairing occurs in the presence of A. We employed different reaction conditions and demonstrated that D-U base-pairing is selective and occurs with A in the reaction mixture (Figure S11). Furthermore, the reaction conditions we tested for base-pairing of equimolar mixtures of D and U include different scenarios of prebiotic water availability,^36^^,^^37^ ranging from bulk water to dry conditions. One such scenario on an early Earth involves wet/dry cycling in hydrothermal pools,^33^^,^^38^^,^^39^ which are considered to be important for the origin of life. Moreover, some of the reaction conditions that we used might be compatible with meteorite post-accretion events such as aqueous alteration^40^ and hydrous metamorphism.^41^ This is relevant since both D and U have been detected in meteorites,^12^^,^^13^ and the chemical evolution of organic inventory under extraterrestrial conditions is of increasing interest.^42^

**Figure 3:**
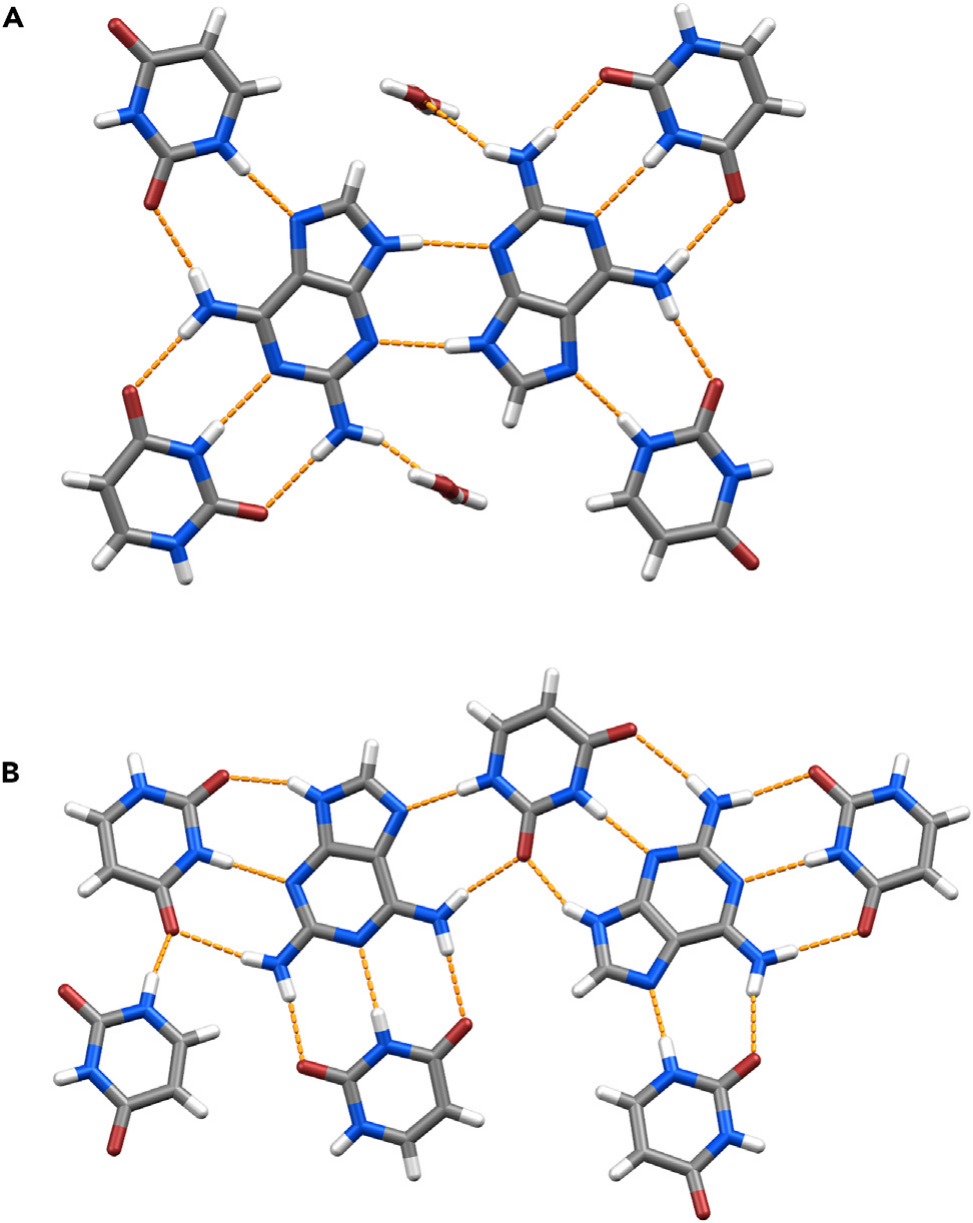
Hydrogen-bond connectivity in D-U cocrystal structures (A) Hydrogen-bonded layers in D-U hyd exhibit D-U and D-D hydrogen bonding. (B) Hydrogen-bonded layers in D-U anhyd exhibit D-U and U-U hydrogen bonding.

To understand the intermolecular interactions of D and U in the solid state, we solved the crystal structures of D-U hyd from SCXRD data (Figure S12) and D-U anhyd from 3D ED data (Figures S13 and S14). D-U hyd is a 1:1 stoichiometry cocrystal channel hydrate whose asymmetric unit comprises D and U molecules assembled by three intermolecular hydrogen bonds and disordered water molecule (Figure S15A). In the crystal, D and U molecules form hydrogen-bonded layers with D-U and D-D hydrogen bonding (Figure 3A). Interestingly, each D molecule forms eight hydrogen bonds, thus involving all possible hydrogen bonding donors and acceptors. Besides filling structural voids and lying in the channels (Figure S15B), the disordered water molecules act as bridging elements connecting adjacent layers through hydrogen bonding (Figure S15C). On the other hand, D-U anhyd is a 1:2 stoichiometry cocrystal anhydrate whose asymmetric unit contains two molecules of U and one molecule of D (Figure S16A). Besides D-U interactions, hydrogen-bonded layers also exhibit homomeric U-U hydrogen bonding interactions (Figure 3B). Similarly, each D molecule forms eight hydrogen bonds, involving all possible hydrogen bonding donors and acceptors. Between the layers, this crystal also exhibits homomeric parallel displaced π-π stacking interactions distanced at 3.7 Å (Figure S16B).

Periodic plane-wave DFT calculations with dispersion corrections were performed on both crystal structures. These simulations confirmed that the fundamental building block for the assembly of D-U hyd and D-U anhyd is the formation of Watson-Crick (W-C) D-U dimers (Table S1), estimated to be around 18 to 20 kcal/mol (in excellent agreement with benchmark quantum-chemical calculations available in the literature^43^). W-C hydrogen bonding is particularly strong in D-U anhyd with N ⋯ N distances as short as 2.67 Å and corresponding interaction energies larger than 20 kcal/mol as taken from periodic DFT relaxed structures. In both D-U hyd and D-U anhyd, we observe the formation of doubly hydrogen bonded D:U structure (intra-layer), especially strong in the hydrated form (shortest N ⋯ N distance approaching 2.74 Å). Layer-to-layer interactions, are typically minor and translate into D:D and U:U π-stacking (D-U anhyd and D-U hyd with a degree of parallel displacement) and also D:U (D-U hyd only). According to periodic DFT, such interactions are typically amounting to ∼ 30 to 50% energy of the corresponding (intra-layer) hydrogen-bonded ones. Formation energies obtained by periodic DFT constraining experimental cells are estimated to be −7.1 kcal/mol for D-U hyd and +2.2 kcal/mol for D-U anhyd, confirming the stability of hydrated form due to the role of water in forming inter-layer hydrogen bonds (Table S2). Besides, in D-U anhyd we observe small distortions from co-planarity. More details on calculation parameters and related analysis can be found in the Experimental Section as well as Tables S1 and S2.

Next, we aimed to study the fate of D-U base pairs under UV irradiation. We investigated the supramolecular arrangement of D-U hyd in an aqueous solution and the sensitivity to UV light with respect to its components. We have performed a series of UVRR measurements using 266 nm as excitation wavelength and *in operando* conditions. Indeed, in our experiment, the aqueous solutions of D, U, and D-U hyd were irradiated continuously with UV light (about 40 mW/cm^-1^), and the resonance Raman spectrum was simultaneously collected from the same scattering volume as a function of the irradiation time. By dissolving the crystalline solid sample in water, we can assume that some hydrogen-bonded interactions between D and U are kept, i.e., in solution there is a dynamical equilibrium between separate D and U and their associated form. While a computational investigation is in progress to quantify such aspects at an atomistic level,^44^ we have included preliminary results showing that several hydrogen-bonded configurations are found at relatively low free energies (see Table S9). Indeed, this appears a sensible element to interpret UVRR results (vide infra).

Although UVRR spectra of both D and U have been investigated,^45^^,^^46^ none of the photodegradation aspects were addressed in those works. On one hand the photostable behavior under UV-light has been associated with a very short excited-state lifetime at the order of picoseconds.^22^ The photostability of uracil has been interpreted with rapid radiationless deactivation from $\pi \pi^{*}$ state $\left(S_{2}\right)$ involving a conical intersection (CI) along a coordinate corresponding to the twist around the double C5C6 bonds.^47^^,^^48^ Very recent works, however, also clarified the existence of a pathway involving a longer-living trapping into $\pi^{*}$ $\left(S_{1}\right)$.^49^ On the other hand, the complex nucleobase photoreactivity, particularly pyrimidine photohydration^25^ and photodimerizations,^24^ have been studied in the last decades. In nucleobases such as U, the formation of CPD in (frozen or liquid) aqueous solutions is a general mechanism that has been widely explored in experiments^24^^,^^50^^,^^51^ and simulations.^52^^,^^53^^,^^54^^,^^55^^,^^56^

**Figure 4:**
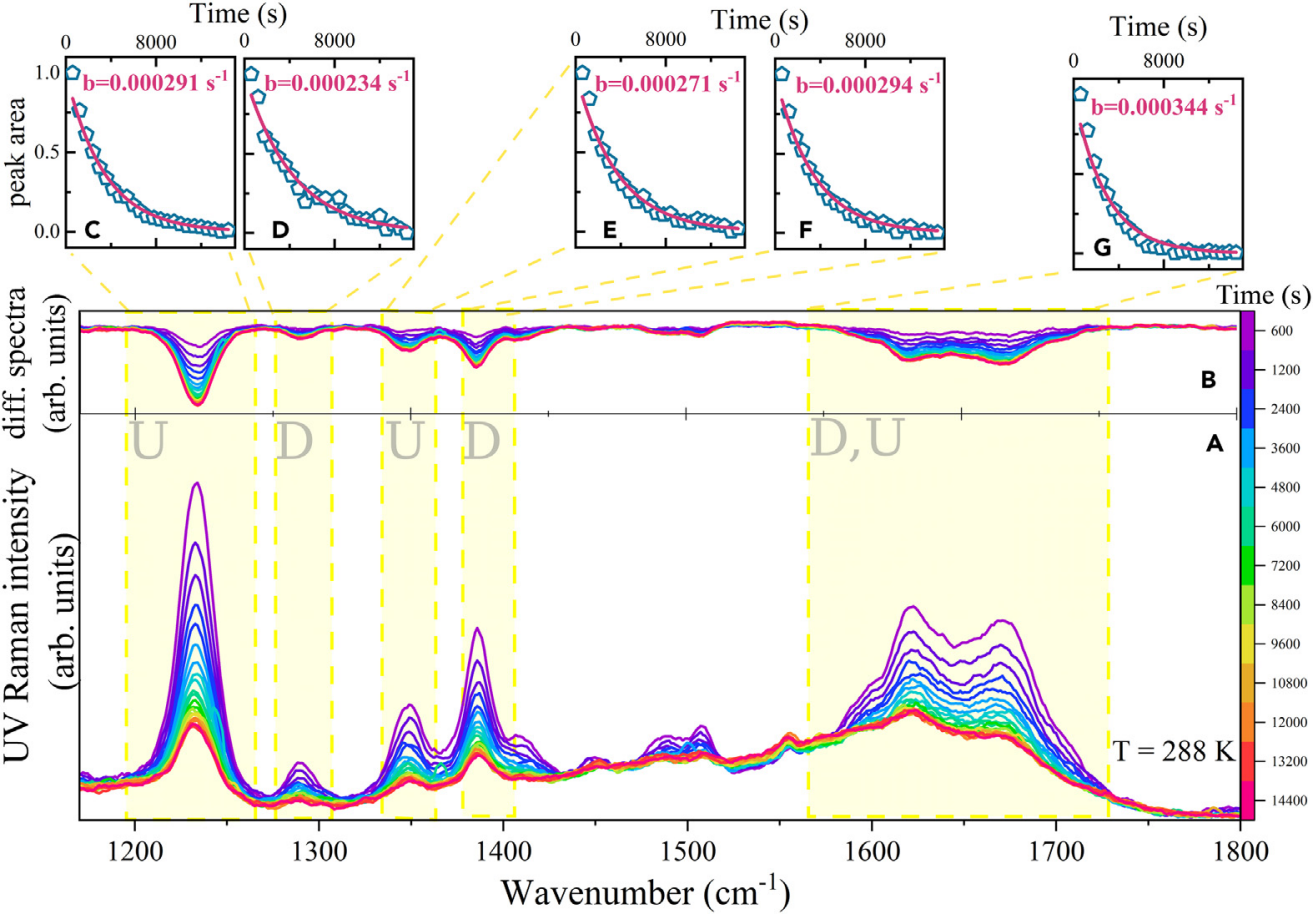
UVRR measurements on D-U solutions upon continuous irradiation (A and B) Effect of UV irradiation directly measured via UV resonance Raman spectroscopy at 266 nm for D-U aqueous solution, collecting one spectrum each 10′ of exposure time (time is traced by the color scale on the right side) at 288 K, measured as intensity over time (A) or as a difference (B). Upper insets (C–G): time traces and monoexponential fitting coefficient of spectral regions highlighted in yellow. Details are provided in Tables S3 and S4.

Interestingly, our results point to negligible effects observed in pure U aqueous solutions (Figure S17). On the other hand, for D (Figure S18) and especially D-U hyd (Figure 4) aqueous solutions at 288 K, we observed a systematic decrease of the signal that we possibly relate to a photodegradation pathway.^57^ Samples were prepared from crystalline compounds dissolved in pure water, with concentrations around 3–6 $\cdot 10^{-4}$ M providing good scattering profiles. We performed a set of 24 measurements at 288 K, each obtained by collecting UVRR signal for 10’ (minutes) of exposition time and found that D-U hyd showed a decrease in scattering intensities in practically all vibrational peaks (Figure 4). We have focused on a few wavenumber intervals and fit the time traces with monoexponential decay profiles of the kind $I=I_{0}\;\exp\left(-bt\right)$ that reproduced the experimental trends reasonably well. It appears that U components in D-U hyd feature a slightly faster decay, and in general, the D-U hyd signal decays faster than for pure D (see Table S4 for fit details).

At 266 nm, the system showing the most intense scattering is U, probably due to the best match with resonance conditions. Possibly due to this reason, vibrations that could be attributed to U components also dominate in intensity for D-U hyd spectra (see Table S3 for a list of attributions). Purine nucleobases, especially in the absence of sugar, have been generally considered quite resistant to UV light.^58^^,^^59^ Photodegredation experiments of 2′-deoxyadenosine and 2,6-diaminopurine 2′-deoxyriboside show that the former is more stable than the latter.^14^ Recently, Caldero-Rodriguez et al.^60^ have been investigating D photochemistry and photophysics, concluding that its behavior follows a fast non-radiative deactivation and/or a weak fluorescence essentially, while only 2% of D degrades under 287 nm light. Our results show that D is less resistant than expected. On the other hand, we expected U to manifest a photoreactive tendency, but we only observed this in the D-U assemblies. It is also important to stress that there is no full consensus about the results of the photodimerization reactions obtained by many groups through the years.^51^ In their attempt to solve the inconsistencies on the interpretation on the photoproduct yields, Shetlar and Basus reported that CPD product was observed as primary dimerization product in either frozen or liquid solutions (in water with and without sensitizers, as well as in organic solvents) and that photodimerization secondary product, the 6–4 adduct, could only be isolated in frozen solutions (approx 9% of yield).^51^ However, the yields and the proportions of the different CPD isomers may vary a lot^24^—suggesting that these processes are all strongly dependent on a set of experimental conditions and the details of the underlying mechanisms.

**Figure 5:**
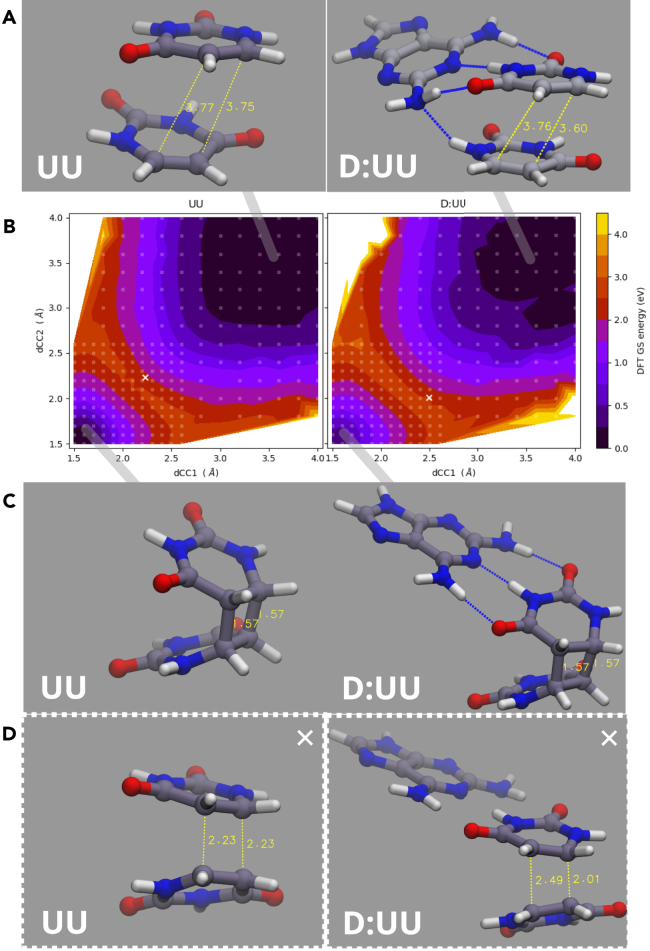
Comparison of photochemical dimerization mechanism in pure U and as hypothesized in the presence of D (A) UU and D:UU π-stacking dimer and (C) UU covalent CPD and D:UU covalent CPD structures obtained as ground state minima ω B97XD DFT (details in Table S5). dCC1 and dCC2 are shown in yellow. (B) Ground state ω B97XD DFT potential energy constructed as relaxed scan along two relevant CC coordinates: dCC1, dCC2 (the grid of evaluated points is shaded). The region where the CI (at CASSCF) were found is marked with a white cross. (D) Structures identified at the conical intersections via CASSCF(4,4) and CASSCF (with dCC1 and dCC2 corresponding values marked in yellow).

To understand the effects of D-U base-pairing on the photodestruction of U, we analyze both U and D-U potential energy surfaces (PESs) in the ground and excited state. As a photoreactive path, we are here focusing on the cyclodimerization (formation of CPD). With respect to the amount of irradiated light, this reaction is characterized by the approaching of a photosteady state,^24^ in agreement with our results collected as a function of time. We will not account for U photohydration reaction, which is known to be reversible and with negligible yields at pH > ∼ 6, as well as 6,4 additions (secondary dimerization).^24^ Besides, photohydration could be ruled out by the fact that we do not observe any effects on solutions of pure U in water. A combination of two coordinates is known to be relevant for the formation of U CPDs, namely the one involving C5 and C6 intra-stack coordinates. We have decided to approach our computational investigation by probing the shape of the PES with respect to these two coordinates as well as searching possible conical intersections (CI), typically involved in such photochemical pathways. We only focus here on the *face-to-face* arrangements of uracil dimers since it has been recently suggested to be the largely dominating photoreactive reactive channel for CPD.^55^ Two coordinates were defined: d(C5C6′) and d(C6C5′), respectively, labeled dCC1 and dCC2. PESs were built in a vacuum, for both the case of two U stacking (UU) and two U stacking with a D forming hydrogen bonds with one of the U molecules (D:UU). This is a sensitive choice based on the D-U anhyd crystal structure motifs and the minima found in the preliminary investigation presented in Table S9, but it is also limited by the fact that only one configuration among the several possible is considered. Such constructed ground state DFT relaxed PES at DFT-ω B97XD level of theory, as well as relaxed S_1_ TD-DFT relaxed PES (same functional) are reported in Figures 5B and S19 (see also Table S5). In such systems, the photoreactive PESs feature a CI, whose presence in CPDs has been demonstrated in multiple flavors.^54^^,^^55^^,^^61^ For both our model systems, we have also performed CASSCF calculations and found that the CI is still encountered regardless of the presence of D. The location of the CIs is also included in Figure 5. Despite its notorious limitations in the description of CI regions and the observed criticalities in the dynamics of nucleobases in the excited state,^49^^,^^62^ DFT, and particularly long-range corrected hybrid functionals, have been employed to describe low-lying excited states of uracil with satisfactory results.^63^^,^^64^ DFT is chosen here for a qualitative description of the static PESs connecting the minima and pointing to the regions where the CI is located, based on CASSCF. According to our molecular orbital (MO) analysis, the nature of the first excited states of U is correctly described (Figures S22–S25; Tables S6 and S7) and compares well with both other functionals (Table S6) and benchmark calculations.^63^ S_1_ in U is a $n\pi^{*}$ state and is therefore dark, as well as in the D:U U complex, however, in the latter, the first bright state seems to acquire a charge-transfer character (Table S6). The PESs clearly feature the covalent cyclodimer (D:U □ U) and the stacked dimer (D:U (π) U) as two low-energy basins. Interestingly, in our $S_{1}$ relaxed PESs, we found a small barrier from the cyclodimer to the region of the CIs. The presence of D obviously de-symmetrizes the surface and the location of the CI. The main consequence of the hydrogen-bonded D is the possibility of accepting a further hydrogen bond with the U-sandwiched molecules (as shown in Figure 5A). Although these results are in-vacuum, they still suggest how D could effectively promote U photodimerization by forming hydrogen bonds with two U molecules simultaneously. We speculate that this could dynamically translate into longer-living non-covalent structures in the presence of D, hence increasing the probability of photodimerization. Besides the configurational variety implicated in this problem, it has been suggested that triplets may play a role, especially at small concentrations of U, by providing additional photodimerization channels.^24^^,^^65^ In principle, a direct involvement of D in the reaction, or a mechanistic scenario more complex than our model, cannot be excluded. However, exploring all possible photochemical pathways of such D-U assembly in aqueous solutions is beyond the scope of this work, and it is a matter for further investigations.

## Discussion

So far, the complementary base-pairing of free canonical nucleobases like U, under the prebiotic conditions, has only been hypothesized. However, our results, which include *in situ* synchrotron PXRD monitoring, show that canonical U base pairs with non-canonical D under the reaction conditions of different water availability and temperatures. Some reaction conditions compatible with D-U base-pairing might have been available on early Earth or extraterrestrial bodies, for example, by wet/dry cycling in hydrothermal land-based pools or under extraterrestrial conditions of meteorite alteration events. We solved the crystal structures of the resulting hydrated and anhydrated D-U base-paired cocrystal assemblies from SCXRD and 3D ED data. Furthermore, we have found experimentally by *in situ* UVRR spectroscopy that D-U aqueous solutions undergo photochemical degradation under 266 nm light, while pure U aqueous solutions do not. Our quantum-chemical and DFT simulations, although performed on a single configuration and therefore hardly conclusive, point to the ability of D to form hydrogen bonds with two U molecules in a π-stacking configuration—hence promoting the photoreaction to CPDs. Our combined state-of-the-art experimental and theoretical studies confirm how the stability of canonical nucleobases depends on many environmental factors and how base-pairing of free nucleobases may affect their photostability.

High-yielding prebiotic syntheses of nucleosides and nucleotides, which bypass nucleobases and form glycosidic bonds on their precursors, are well known in the literature.^15^^,^^66^^,^^67^^,^^68^ On the other hand, non-covalent interactions between small building blocks of life, such as nucleobases, may provide new insights into chemical evolution. In particular, intermolecular interactions between nucleobases can significantly alter their (already complex) potential energy landscapes and dynamically open the possibility to photoactivation-driven reactions. In the early stages of chemical evolution, nucleobases with complementary supramolecular interactions may have been selected or neglected based on their thermodynamic and kinetic stability or photostability.^11^^,^^22^

### Limitations of the study

We acknowledge the complexity of the photodegradation of the D-U base-pair. In this study, we were not able to investigate all possible photochemical pathways of the whole configuration space and neither to further characterize experimentally the photoproducts. These aspects are a matter for further investigation.

## STAR★Methods

### Key resources table

**Table:** 

REAGENT or RESOURCE	SOURCE	IDENTIFIER
**Chemicals**		

Uracil	Carbosynth	CAS number 66-22-8
2,6-diaminopurine	Carbosynth	CAS number 1904-98-9

**Deposited data**		

D-U hyd	This paper	CCDC deposition number 2059340
D-U anhyd	This paper	CCDC deposition number 2247776

**Software and algorithms**		

TOPAS	Bruker	Bruker-AKS Karlsruhe^69^
CrysAlis^*PRO*^	Rigaku	Diffraction^70^
PLATON	Open source	Spek^71^
ORTEP-3	Open source	Farrugia^72^
Mercury	CCDC	Macrae et al.^73^
Olex2	Open source	Dolomanov et al.^74^
Quantum Espresso	Open source	Giannozzi et al.^75^
Gaussian	Gaussian, Inc.	https://gaussian.com/

### Resource availability

#### Lead contact

Further information and requests for resources and reagents should be directed to and will be fulfilled by the lead contact, Dr Tomislav Stolar (tomislav.stolar@gmail.com).

#### Materials availability

This study did not generate new unique reagents. All product materials were obtained as described below. Further information and requests can be addressed to lead contact.

#### Data and code availability

• Computational relevant data such as coordinates obtained from proprietary codes are published at the end of the Supporting information. Coordinates obtained from open-source codes (crystal-related structural analysis performed with Quantum-Espresso) can be obtained following the reproducible protocol detailed in the Periodic DFT section and in the Supporting Information, starting from the experimental cystallographic structure as input. Crystallographic data are available free of charge from the CCDC (deposition numbers 2059340 and 2247776).
• This paper does not report original code.
• Any additional information required to reanalyze the data reported in this paper is available from the lead contact upon request.

### Method details

#### Materials and characterization

Uracil (U) and 2,6-diaminopurine (D) were purchased from Carbosynth. Grinding experiments were conducted in 14 mL poly(methyl methacrylate) (PMMA) containers using InSolido Technologies vibratory ball mill. Two 1.4 g stainless steel balls (7 mm in diameter) were used as milling media and the reactions were conducted at a 30 Hz frequency. In grinding reactions with H_2_O, 20 μL of H_2_O was added to the overall 200 mg of the reaction mixture (1.5:1 ratio of H_2_O to D and U). Grinding under low-temperature conditions was performed using liquid nitrogen. PMMA jars containing the reaction mixture were immersed in liquid nitrogen before mounting on the mill and liquid nitrogen was poured continuously over the jars during 30 min of ball milling. Slurry reactions were performed by stirring an aqueous suspension of D and U (equimolar amounts of D and U, overall mass of the reaction mixture 100 mg) in 3 mL of distilled H_2_O at room temperature in a 10 mL round bottom flask. Aging reactions were conducted by placing a plastic tube containing a solid sample in a glass bottle whose bottom was filled with distilled H_2_O. The aging setup was a closed system and the glass bottle was closed with a cap. Ex situ PXRD data were collected on Panalytical Aeris tabletop X-ray diffractometer, with Cu*K*_α_ radiation (40 kV, 7.5 mA) in Bragg-Brentano geometry, with the sample mounted on a zero background silicon plate. TGA and DSC experiments were performed by Simultaneous thermal analyzer (STA) 6000 (PerkinElmer, Inc.) in platinum crucibles at 5 °C min^-1^ heating rate under nitrogen gas purging at a flow of 20 mL min^-1^. Rietveld refinement^76^ was performed in TOPAS.^69^

#### *In situ* synchrotron PXRD monitoring of grinding and wet/dry cycling

*In situ* monitoring of grinding D and U with H_2_O was performed at the P02.1 beamline at PETRA III, DESY (Hamburg, Germany). 20 μL of H_2_O was added to the overall 200 mg of the equimolar D and U reaction mixture (1.5:1 ratio of H_2_O to D and U). The X-ray beam (λ = 0.20741 Å) was set to pass through the bottom of the PMMA reaction vessel. Exposure time was set to 10 s. Diffraction data were collected on a PerkinElmer XRD1621 flat-panel detector positioned 1595 mm from the sample, which consisted of an amorphous Si sensor equipped with a CsI scintillator (pixel number: 2048 × 2048, pixel size: 200 × 200 $m^{2}$). To obtain the classic one-dimensional PXRD pattern, the two-dimensional diffraction images were integrated with the DAWN Science package.

Variable-temperature PXRD for wet/dry cycling of D-U hyd was performed at P02.1 beamline at PETRA III, DESY (Hamburg, Germany). A capillary of 0.5 mm width was filled with ca. 5 mg of D-U hyd sample and was heated using Oxford Cryostream system (operating in 100–500 K temperature range). The distance between the sample and the detector was 2010 mm. X-rays of 0.20741 Å wavelength were used, and X-ray diffraction patterns were collected on a spinning capillary. The sample was thermally equilibrated at 300 K and was heated using a 5 K/min ramp rate. X-ray diffraction patterns were collected in 10 K steps. After reaching the desired temperature set-point, the sample was thermally equilibrated, and X-ray diffraction pattern was collected. The exposure time for each collected diffraction pattern was 60 s for the dark image plus 60 s for the real image. To obtain 2D plots for *in situ* synchrotron PXRD monitoring, data were processed in MATLAB.

#### Single-crystal X-Ray diffraction

To obtain single crystals of D-U hyd, 15 mg of a dry ground equimolar mixture of D and U was dissolved in 5 mL of methanol. Single-crystals were grown after solvent evaporation at room temperature. Single crystal measurements were performed on an Oxford Diffraction Xcalibur Nova R (microfocus Cu tube) equipped with an Oxford Instruments CryoJet liquid nitrogen cooling device. Program package CrysAlis^*PRO*^^70^ was used for data reduction and numerical absorption correction. The structures was solved using SHELXS97 and refined with SHELXL-2017.^77^ The model was refined using the full-matrix least-squares refinement; all non-hydrogen atoms were refined anisotropically. Hydrogen atoms were located in a difference Fourier map and refined as a mixture of free-restrained and riding entities. The co-crystallized water molecule (O3) is disordered over two positions (designated as A and B) with respective occupancies of 63% and 37%. Molecular geometry calculations were performed by PLATON^71^ and molecular graphics were prepared using ORTEP-3,^72^ and Mercury.^73^ Crystallographic and refinement data for D-U hyd is shown in Figure S15. The structure has been deposited in the Cambridge Crystallographic Data Center (CCDC) as no. 2059340.

#### 3D electron diffraction

ED measurements for D-U anhyd were collected using the Rigaku XtaLAB Synergy-ED, equipped with a Rigaku HyPix-ED detector optimized for operation in the continuous rotation 3D-ED experimental setup.^78^ Data acquisition was performed at ambient temperature under high vacuum with an electron wavelength of 0.0251 Å. The instrument was operated and the diffraction data were processed in the program CrysAlis^*PRO*^.^79^ A multi-scan absorption correction was performed using spherical harmonics implemented in SCALE3 ABSPACK scaling algorithm in CrysAlis^*PRO*^. The structure was solved using ShelXT,^77^ and subsequently, refined with kinematical approximation using ShelXL^80^ in the crystallographic program suite Olex2.^74^^,^^81^ By merging data of three individual grains/datasets, completeness of 96.2% up to a resolution of 0.80 Å was achieved. Non-hydrogen atoms were assigned anisotropic displacement parameters unless stated otherwise. The hydrogen atoms bonded to nitrogen atoms were located from Fourier difference maps. Other hydrogen atoms were placed in idealized positions and included as riding. Isotropic displacement parameters for all hydrogen atoms were constrained to multiples of the equivalent displacement parameters of their parent atoms with $U_{iso}$ (H) = 1.2 $U_{eq}$ (parent atom). Enhanced rigid bond restraints^82^ with standard uncertainties of 0.001 Å^2^ was applied. The experimental and refinement details are given in Figure S16. The crystal structure of D-U anhyd has been deposited in CCDC as no. 2247776.

#### Periodic DFT simulations

Periodic plane-wave DFT calculations were performed with Quantum Espresso^83^^,^^75^ using GBRV pseudopotentials^84^ and vdW-DF-cx^85^^,^^86^ exchange-correlation functional that is well suited for this type of systems.^87^^,^^88^^,^^89^^,^^90^ The plane-wave basis set cutoff is 600 eV, and the first Brillouin zone is sampled by the Monkhorst–Pack k-point mesh with a density of 5 Å. Coordinates from experimental XRD were relaxed according to BFGS algorithm, first by keeping cell parameters equal to experimental, and then further relaxed allowing for combined unit cell and positions relaxations. From these two DFT calculations (0 K) minimization, we respectively obtained *rigid-cell formation energy*:

$$E_{rigid\;cell}^{form}\left(D-U\;hyd\right)={E\left(D-U\;hyd\right)}^{\exp.cell}-\left[{E\left(D\;hyd\right)}^{\exp.cell}+{E\left(U\right)}^{\exp.cell}\right]$$

$$E_{rigid\;cell}^{form}\left(D-U\;anhyd\right)={E\left(D-U\;anhyd\right)}^{\exp.cell}-[{E\left(D\;hyd\right)}^{\exp.cell}+{E\left(U\right)}^{\exp.cell}-{E\left(H_{2}O\;Ih\;ice\right)}^{\exp.cell}$$

and *full formation energy*:

$$E_{full}^{form}\left(D-U\;hyd\right)={E\left(D-U\;hyd\right)}^{relaxed\;cell}-\left[{E\left(D\;hyd\right)}^{relaxed\;cell}+{E\left(U\right)}^{relaxed\;cell}\right]$$

$$E_{full}^{form}\left(D-U\;anhyd\right)={E\left(D-U\;anhyd\right)}^{relaxed\;cell}-[{E\left(D\;hyd\right)}^{relaxed\;cell}+{E\left(U\right)}^{relaxed\;cell}-{E\left(H_{2}O\;Ih\;ice\right)}^{relaxed\;cell}$$

where each energy term embeds factors to compare the different unit-cell stoichiometries. These results are reported in Table S1. For in-vacuum complexes and interaction energy $V_{AB}$ (Table S2), results were obtained using periodic DFT by relaxing clusters obtained from crystal placed in a large unit cell (box size of 70. $a_{0}$ ), so to minimize interaction between periodic images. The usage of planewave-DFT avoid any superposition errors in estimating the interaction energies in molecular clusters. Periodic DFT simulations were run at the high-performing computing cluster of the Division of Theoretical Physics of the Ruđer Bošković Institute, Zagreb, Croatia.

#### UV resonance Raman spectroscopy

Multi-wavelength UVRR spectra have been collected at the BL10.2-IUVS beamline of Elettra Sincrotrone Trieste by exploiting the experimental setup described in detail in Rossi et al.^91^ Different excitation wavelengths in the deep UV range (220–270 nm) were employed. These excitation conditions were chosen to obtain suitable resonance Raman signals for the examined samples and, at the same time, the best features in terms of the spectral resolution and signal-to-noise ratio in the whole concentration range considered. In particular, the reported quantitative information specifically refers to the UVRR spectra acquired using 266 nm as excitation wavelength. The Raman signal was collected in back-scattered geometry using a single pass of a Czerny-Turner spectrometer (Trivista 557, Princeton Instruments, 750 mm focal length) and detected with a UV-optimized CCD camera. The photochemical degradation of U, D-U, and D systems has been followed by irradiating the sample with a UV laser source at 266 nm and, at the same time, consecutively collecting UVRR spectra of the solution every 10 min for a total time of 4 h. The advantage of the adopted experimental set-up is the possibility to use the same UV excitation source both to induce the photochemical degradation of D-U and to record the UVRR spectra. The spectral resolution was set at 1.2 cm^-1^/pixel. The calibration of the spectrometer was standardized using cyclohexane (spectroscopic grade, Sigma-Aldrich). The power of the beam on the sample was measured to be 0.3–0.4 mW with a spot area of about 1 mm^2^. Solid samples (D, U, and D-U hyd) were dissolved in pure water. Such freshly prepared solutions were placed in suitable suprasil-quartz cuvettes with an optical path of 10 mm. Atmosphere in cuvette was made inert by nitrogen flow, and then measured under a magnetic stirrer. During the Raman measurements, the temperature of the solutions was controlled using a sample holder equipped with a thermal bath coupled to a resistive heating system to keep the temperature of the sample at a fixed value with the stability of ± 0.1° C. The collected UVRR spectra were subtracted from a 2-order polynomial baseline and the cosmic rays were removed. We have assessed the intensity of the bands of interest through an integration algorithm applied to the wavenumber regions of interest.

#### Quantum-chemical and DFT simulations in-vacuum

Ground and excited state 2D PESs for UU and D-UU dimerization were calculated using the *Gaussian09* package^92^ on the Graham cluster operated by Compute Canada. Energies were calculated using the ω B97XD functional^93^ and 6-31G∗ basis set.^94^ 2D PES ground state optimizations involved freezing two carbon-carbon bonds between U and U (using the opt = modredundant keyword) from 1.5 to 4.0 Å. Scans from 1.5 to 2.6 Å were performed with a precision of 0.1 Å, and scans from 2.6 to 4.0 Å were performed with a precision of 0.2 Å. Both unrelaxed and relaxed scans were also performed for the first excited state using TDDFT starting from the ground state geometries. In addition, at the same level of theory, a full ground state minimization was run for each of the basins identified in the PESs for UU and D:UU, followed by TDDFT evaluation of excitation energies and first excited state relaxations. Comparison of our ω B97XD TDDFT results with CAM-B3LYP^63^ is provided in Table S6.Ground-state minima were reoptimized by accounting for the superposition error,^95^ with minimal changes (Table S5). After a proper evaluation of the active spaces, CI optimizations through *opt=conical* via CASSCF(4,4) for UU and CASSCF(6,6) for DU:U and 6-31G∗ basis set using *Gaussian16* package^96^ were successfully performed (details in Table S8). These final CASSCF calculations were run at the Isabella cluster in Zagreb, Croatia.

## Supplemental information

Document S1. Figures S1‒S26, Tables S1–S9, and Data S1
