# Supplementary material for: Base-pairing of uracil and 2,6-diaminopurine: from cocrystals to photoreactivity
Source: iScience. 2024 May 7;27(6):109894. doi: 10.1016/j.isci.2024.109894 (PMC11112615; doi:10.1016/j.isci.2024.109894)
Supplement: Document S1. Figures S1‒S26, Tables S1–S9, and Data S1 [file mmc1.pdf]

iScience, Volume *27*

Supplemental information

Base-pairing of uracil

and 2,6-diaminopurine: from cocrystals

to photoreactivity

Tomislav Stolar, Ben K.D. Pearce, Martin Etter, Khai-Nghi Truong, Tea Ostojić, Andraž Krajnc, Gregor Mali, Barbara Rossi, Krešimir Molčanov, Ivor Lončarić, Ernest Meštrović, Krunoslav Užarević, and Luca Grisanti

Supporting Information for "Base-pairing of uracil and 2,6-diaminopurine: from cocrystals to photoreactivity"

Tomislav Stolar,*,[a],[b] Ben K. D. Pearce,[c] Martin Etter,[d] Khai-Nghi Truong,[e] Tea Ostojić,[a] Andraž Krajnc,[f] Gregor Mali,[f] Barbara Rossi,[g] Krešimir Molčanov,[a] Ivor Lončarić,[a] Ernest Meštrović,*,[h] Krunoslav Užarević,*,[a] and Luca Grisanti*,[a],[i]

[a]Ruđer Bošković Institute, 10000 Zagreb, Croatia

[b]Federal Institute for Materials Research and Testing (BAM), 12489 Berlin, Germany

[c]Johns Hopkins University, Baltimore, MD 21218, USA

[d]Deutsches Elektronen-Synchrotron (DESY), 22607 Hamburg, Germany

[e]Rigaku Europe SE, 63263 Neu-Isenburg, Germany

[f]National Institute of Chemistry, 1000 Ljubljana, Slovenia

[g]Elettra Sincrotrone Trieste, 34149 Trieste, Italy

[h]Faculty of Chemical Engineering and Technology, University of Zagreb, 10000 Zagreb, Croatia

[i]National Research Council - Materials Foundry Institute (CNR-IOM) c/o SISSA (International School for Advanced Studies), 34136 Trieste, Italy

E-mail: tomislav.stolar@gmail.com; emestrov@fkit.hr; krunoslav.uzarevic@irb.hr; luca.grisanti@irb.hr

List of Figures

Fig. S1 PXRD measured for various experimental combination S4
Fig. S2 PXRD refinement of D U S5
Fig. S3 PXRD measured at various stage of the preparation S5
Fig. S4 In situ PXRD monitoring after grinding. S6
Fig. S5 PXRD refinement during the preparation of D-U hyd S6
Fig. S6 PXRD refinement of heated D-U (410K) S7
Fig. S7 PXRD refinement of heated D-U (490K) S7
Fig. S8 Calorimetric characterisation S7
Fig. S9 PXRD refinement of heated D-U (530K) S8
Fig. S10 CPMAS NMR of D-U hyd after heating S8
Fig. S11 PXRD measured combining different nucleobases S9
Fig. S12 Summary of experimental single-crystal crystallographic data S10
Fig. S13 Summary of experimental single-crystal crystallographic data S11
Fig. S14 Images from 3D ED experiments S12
Fig. S15 D-U hyd crystal structure S13
Fig. S16 D-U anhyd crystal structure S14
Fig. S17 UVRR measurements on U solutions upon continuous irradiation S15
Fig. S18 UVRR measurements on D solutions upon continuous irradiation S16
Fig. S19 TDDFT relaxed PES of S1 for UU and DUU S17
Fig. S20 H-bonded N(D)-N(U) distance S18
Fig. S21 Unexpected S_1 relaxed UU structure showing proton transfer S18
Fig. S22 MOs for isolated D and U S19
Fig. S23 MOs for UU structures S20
Fig. S24 MOs for DUU structures S21
Fig. S25 MOs for DUU structures S22
Fig. S26 Free energy surface of U and D in water - preliminary results S23

List of Tables

Table S1 Analysis of interactions from crystal structures S24

Table S2 Crystal formation energies S24

Table S3 UVRR peak assignements S25

Table S4 UVRR decay fit details and parameters S25

Table S5 In-vacuum DFT energies and geometry information S26

Table S6 TDDFT transitions: energies and oscillator strenghts S27

Table S7 Contribution to each TDDFT transition S28

Table S8 Details for CASSCF calculations for identifying the conical intersections S29

Table S9 Classification of free-energy minima of D and U. S30

List of Data

Data S1 **S31**

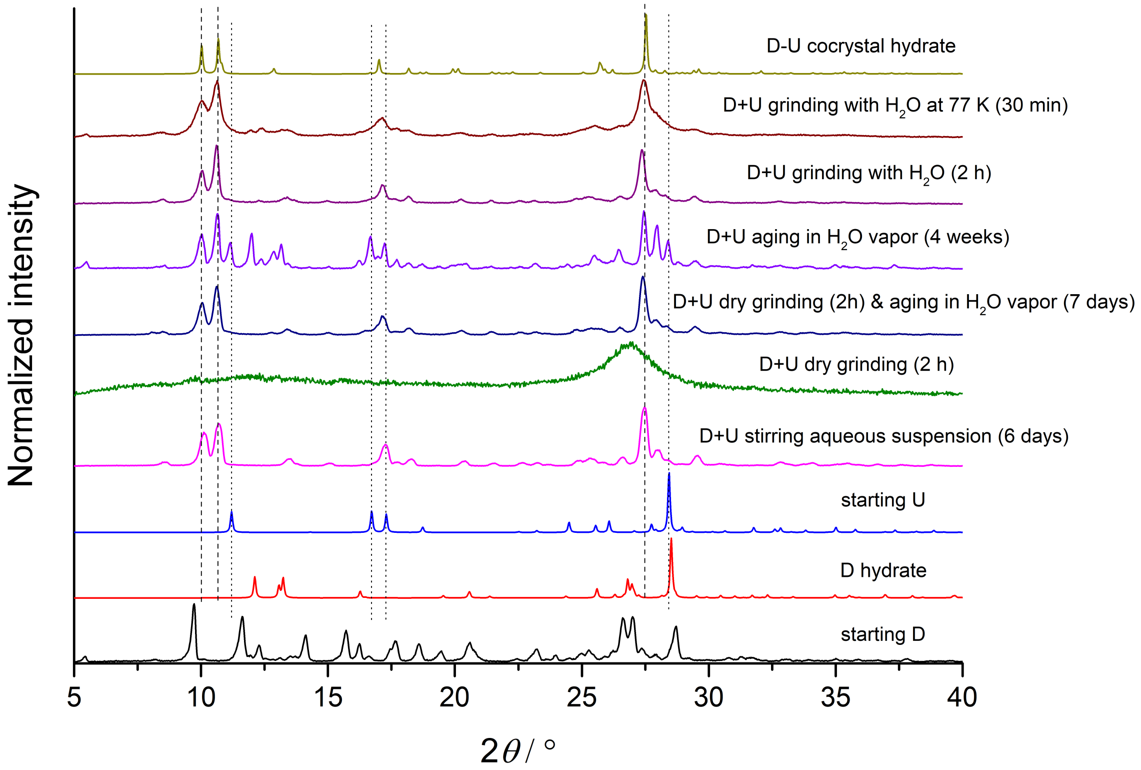

Figure S1: **PXRD measured for various experimental combination .** PXRD patterns for the reactants and products of different reactions. Most prominent peaks corresponding to reactants are highlighted with dotted lines, whereas those corresponding to **D-U hyd** are highlighted with dashed lines. PXRD patterns of D hydrate (DOI: 10.1021/cg500602x) and **D-U hyd** (this work) are simulated from CIFs. Note that D corresponds to 2,6-diaminopurine and U to uracil.

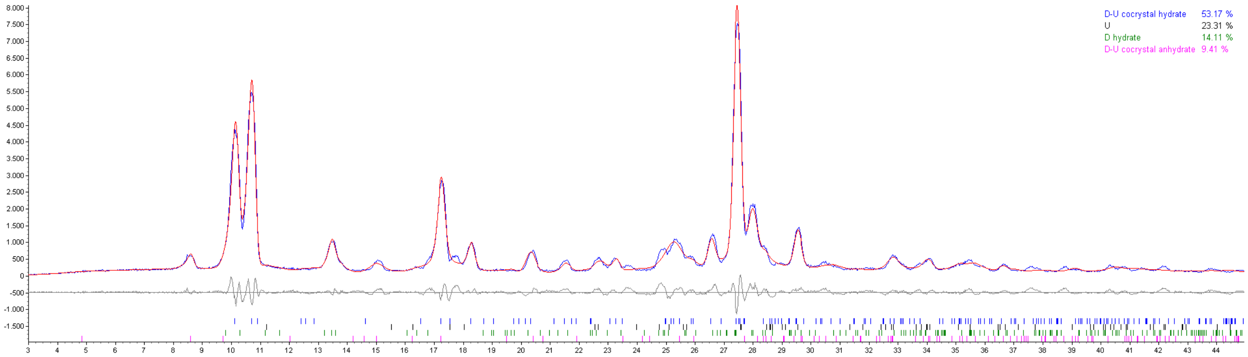

Figure S2: **PXRD refinement of D U.** Rietveld refinement of a PXRD pattern that was obtained after stirring an aqueous suspension of D and U for six days at room temperature. The following CIFs were used: D hydrate (DOI: 10.1021/cg500602x) and **D-U hyd** (this work) and **D-U anhyd** (this work). The relative weight fractions are given in the right top corner. The major component of the solid sample was **D-U hyd**.

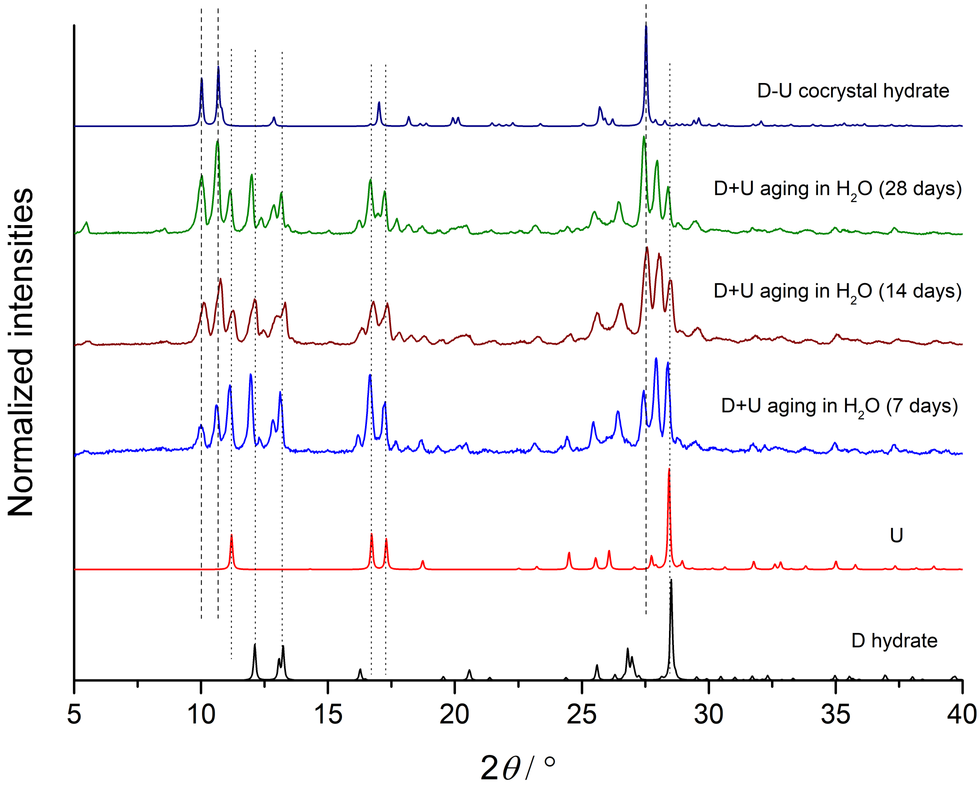

Figure S3: **PXRD measured at various stage of the preparation.** PXRD patterns for the reactants and products of aging reactions on different time scales. Most prominent peaks corresponding to reactants are highlighted with dotted lines, whereas those corresponding to **D-U hyd** are highlighted with dashed lines.

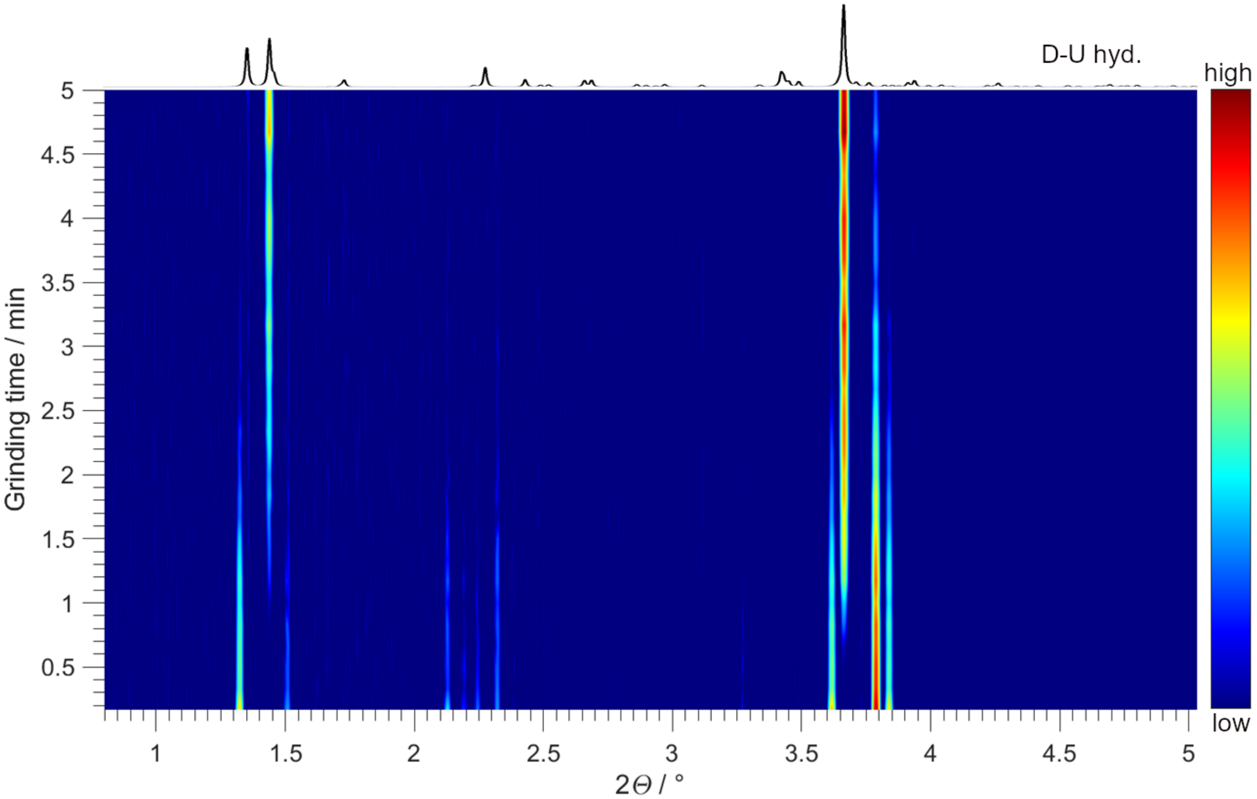

Figure S4: **In situ PXRD monitoring after grinding..** *In situ* monitoring of grinding 200 mg of equimolar amount of D and U with 20 μL of H_2O by synchrotron PXRD ($\lambda = 0.20741$ Å). **D-U hyd** started to form in the first minute of grinding. Simulated PXRD pattern of **D-U hyd** is given at the top.

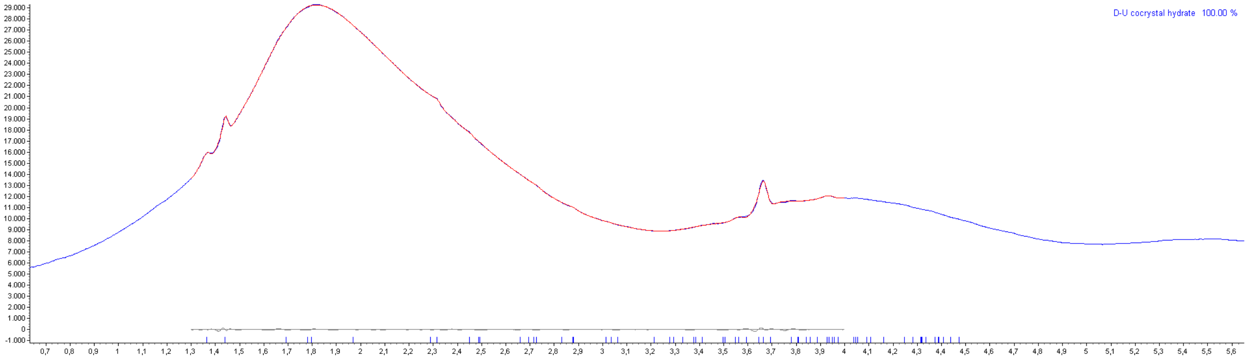

Figure S5: **PXRD refinement during the preparation of D-U hyd.** Rietveld refinement of a PXRD pattern that was obtained by *in situ* synchrotron PXRD monitoring after 30 min of grinding 200 mg of equimolar amount of D and U with 20 μL of H_2O. The sample was confirmed to correspond to **D-U hyd**.

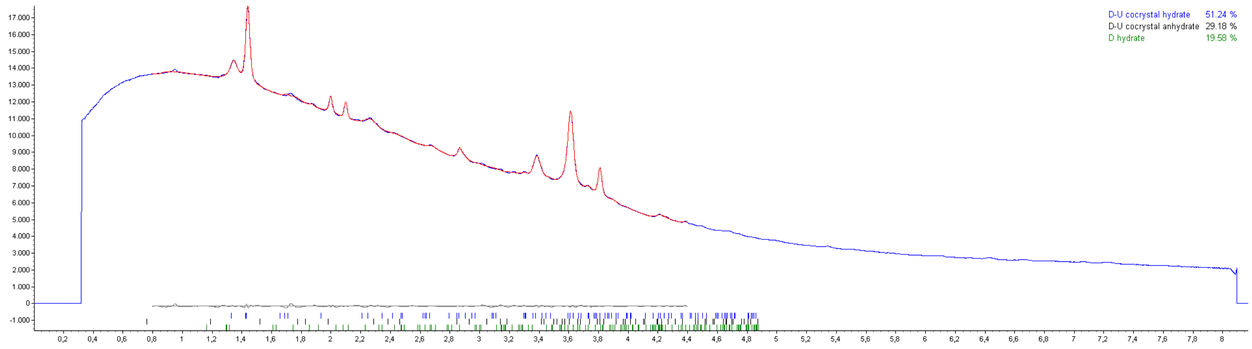

Figure S6: **PXRD refinement of heated D-U (410K).** Rietveld refinement of a PXRD pattern that was obtained by *in situ* synchrotron PXRD monitoring after heating **D-U hyd** to 410 K.

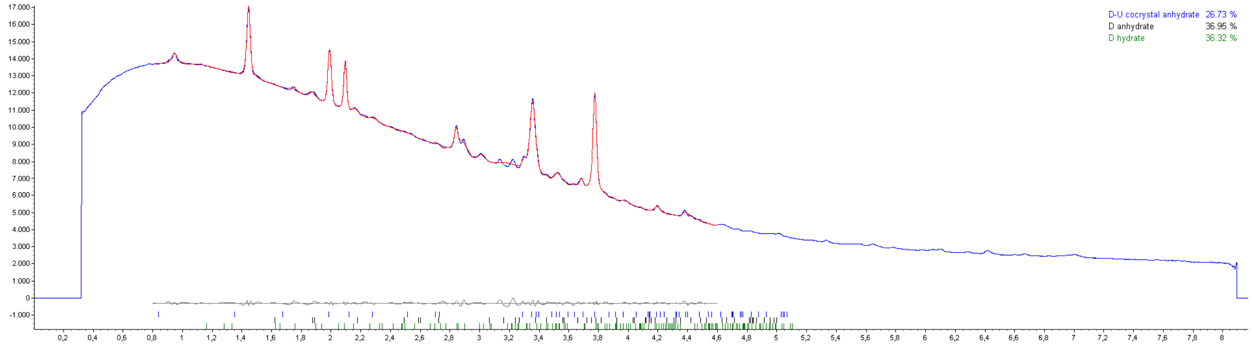

Figure S7: **PXRD refinement of heated D-U (490K).** Rietveld refinement of a PXRD pattern that was obtained by *in situ* synchrotron PXRD monitoring after heating **D-U hyd** to 490 K.

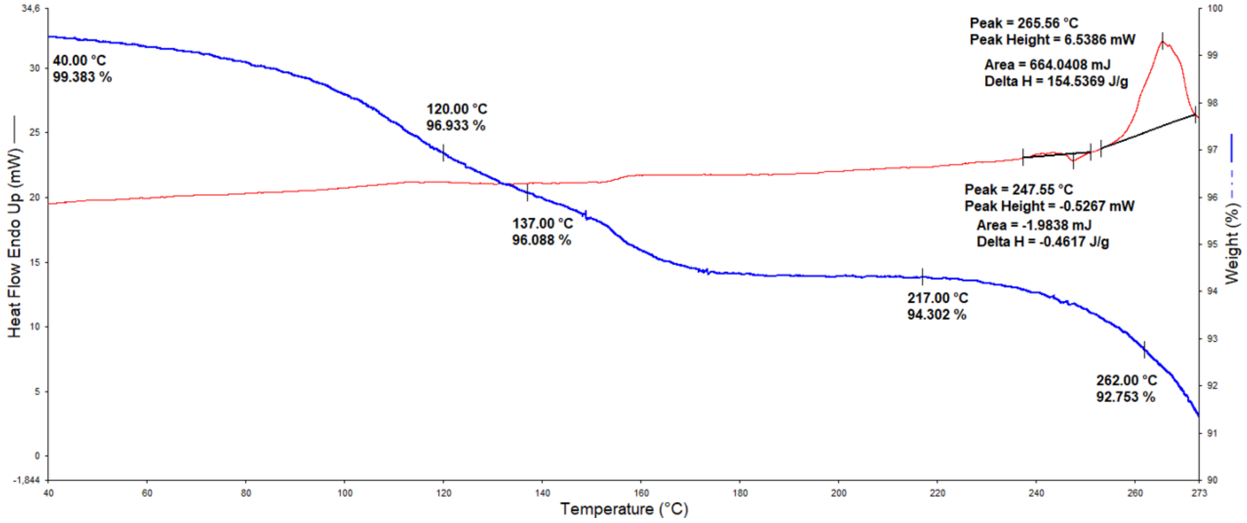

Figure S8: **Calorimetric characterisation.** DSC and TGA curves for heating **D-U hyd**.

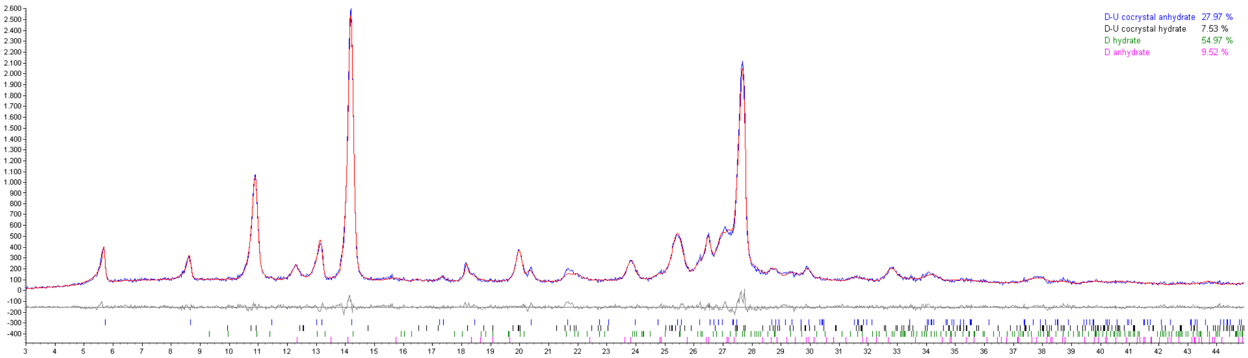

Figure S9: **PXRD refinement of heated D-U (530K).** Rietveld refinement of a PXRD pattern that was obtained from the **D-U hyd** sample heated in a DSC experiment from the Figure S11 to 530 K.

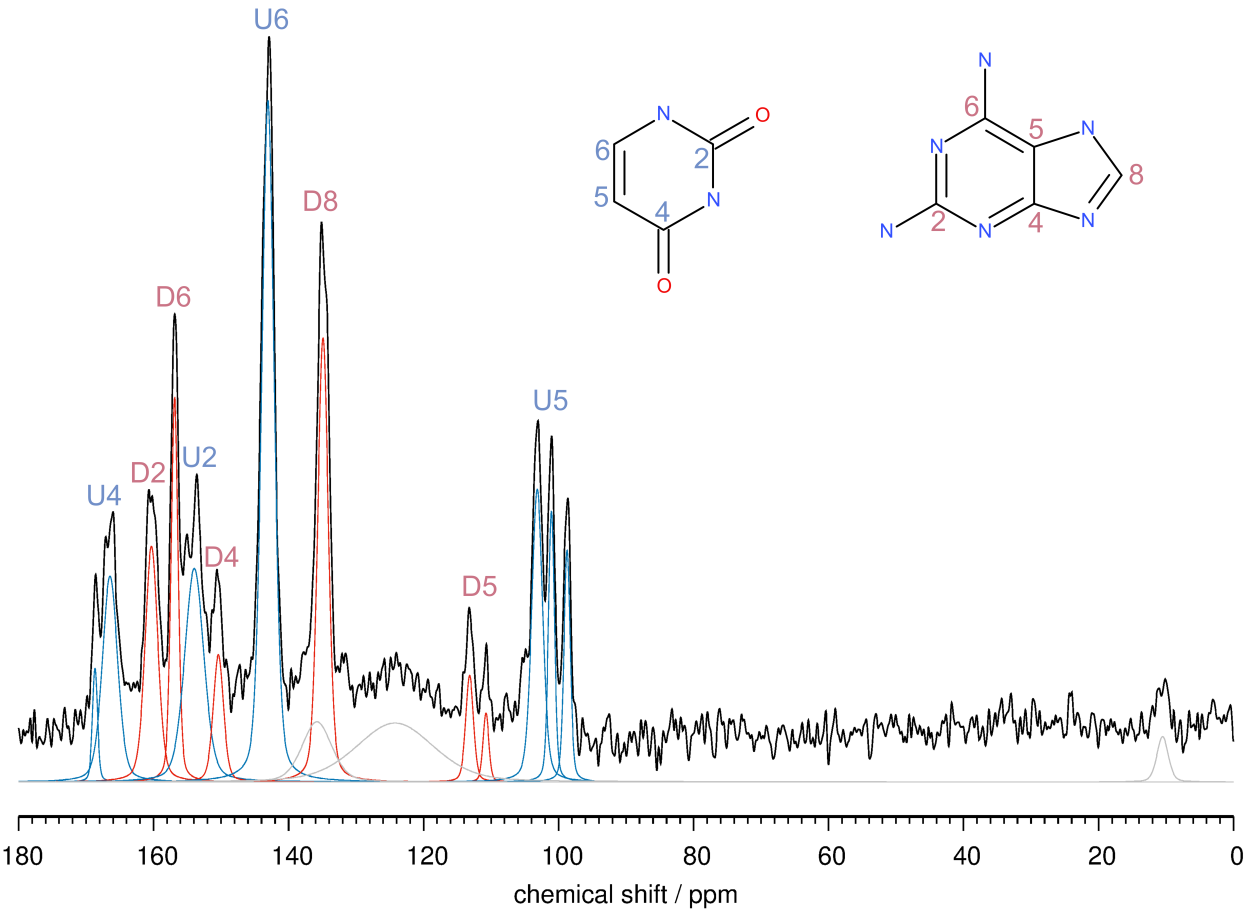

Figure S10: **CPMAS NMR of D-U hyd after heating.** Assigned ^{1}H-^{13}C cross-polarization magic-angle spinning (CPMAS) nuclear magnetic resonance spectra after heating **D-U hyd** in a DSC experiment from the Figure S9 to 530 K. The sample was not phase pure. In the ^{1}H-^{13}C CPMAS experiments, a cross-polarization (CP) block of 5 ms, and signal acquisition with high-power XiX proton decoupling were used, with a repetition delay of 28 s and a total of 6,000 scans collected. ^{13}C chemical shift axis was referenced relative to the signal of tetramethylsilane.

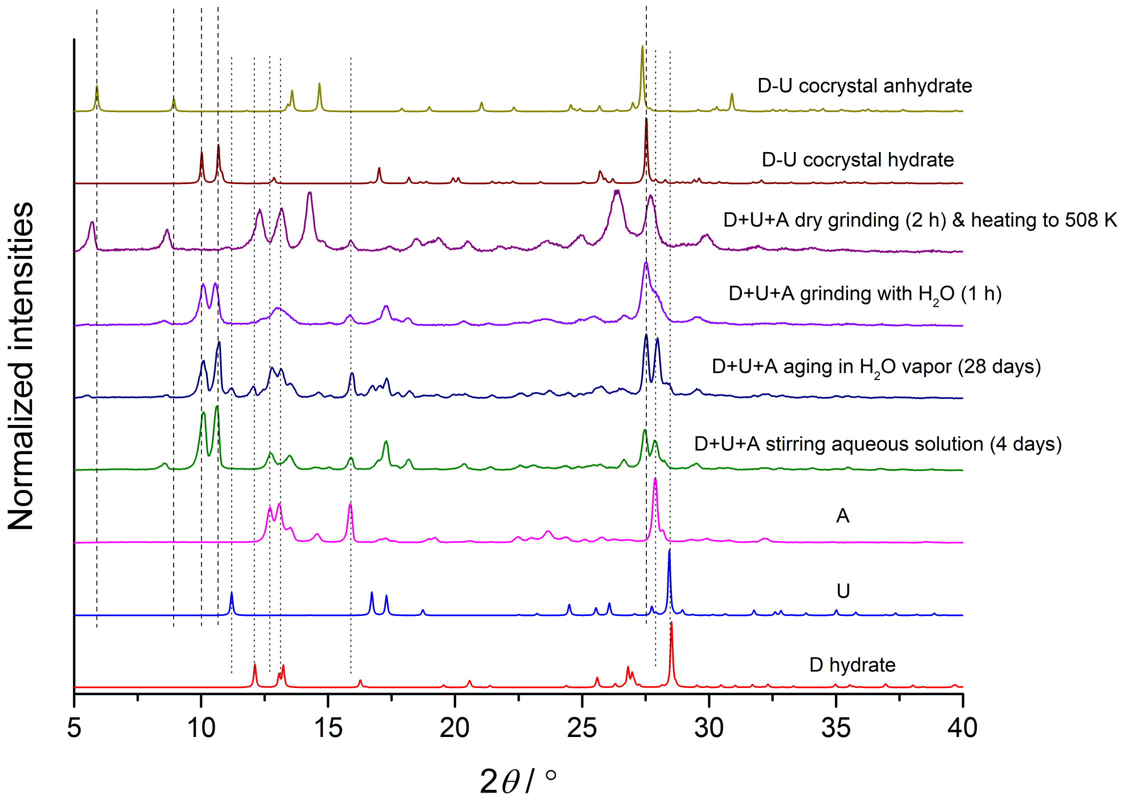

Figure S11: **PXRD measured combining different nucleobases .** PXRD patterns for the selectivity experiements (D and U base-pairing in the presence of adenine (A)). Most prominent peaks corresponding to reactants are highlighted with dotted lines, whereas those corresponding to **D-U hyd** and **D-U anhyd** are highlighted with dashed lines. PXRD patterns of D hydrate (DOI: 10.1021/cg500602x), **D-U hyd** (this work), and **D-U anhyd** (this work) are simulated from CIFs.

Compound	**D-U cocrystal hydrate**
Empirical formula	$C_9H_{12}N_8O_3$
Formula wt. / g mol^{-1}	280.27
Colour	colorless
Crystal dimensions / mm	0.18 x 0.12 x 0.07
Space group	*C* 2/*c*
a / Å	10.6851(8)
b / Å	16.5477(10)
c / Å	14.2161(12)
α / °	90
β / °	102.930(8)
γ / °	90
Z	8
V / Å^3	2449.9(3)
D_{calc} / g cm^{-3}	1.520
λ / Å	1.54719 (Cu*Kα*)
μ / mm^{-1}	1.017
Θ range / °	5.02 – 76.84
T / K	293(2)
Diffractometer type	Xcalibur Nova
Range of *h*, *k*, *l*	$11 < h < 13$; $-20 < k < 17$; $-16 < l < 17$
Reflections collected	5096
Independent reflections	2498
Observed reflections ($I \geq 2\sigma$)	1785
Absorption correction	Multi-scan
T_{min}, T_{max}	0.4930, 1.0000
R_{int}	0.0380
R (F)	0.0673
R_w (F^2)	0.2133
Goodness of fit	1.012
H atom treatment	Mixed
No. of parameters	210
No. of restraints	12
$\Delta\rho_{max}$, $\Delta\rho_{min}$ (eÅ^{-3})	0.278; - 0.244

Figure S12: **Summary of experimental single-crystal crystallographic data.** Crystallographic, data collection and refinement data for single crystal X-ray diffraction. The structure has been deposited in CCDC as no. 2059340.

Crystal data for **D-U cocrystal anhydrate**: CCDC 2247776, colorless plate, $C_{13}H_{14}N_{10}O_4$, M_r = 374.34 gmol^{-1}, monoclinic, space group *Pn* (No. 7), a = 3.69(13) Å, b = 14.98(14) Å, c = 13.24 (13) Å, α = 90°, β = 94.9(4)°, γ = 90°, V = 729(27) Å^3, Z = 2, Z' = 1, T = 298 K, *m*(transmission electron microscope) = 0.000, 8595 total reflections, 2554 with $I_0 > 2\sigma(I_0)$, R_{int} = 0.1018, 2820 data, 245 parameters, 197 restraints, GoF = 2.561, R_1 = 0.1318 and wR_2 = 0.3396 [$I_0 > 2\sigma(I_0)$], R_1 = 0.1506 and wR_2 = 0.3647 (all reflections), 0.228 < dΔρ < -0.231.

Figure S13: **Summary of experimental single-crystal crystallographic data.** Crystallographic, data collection and refinement data for 3D electron diffraction.

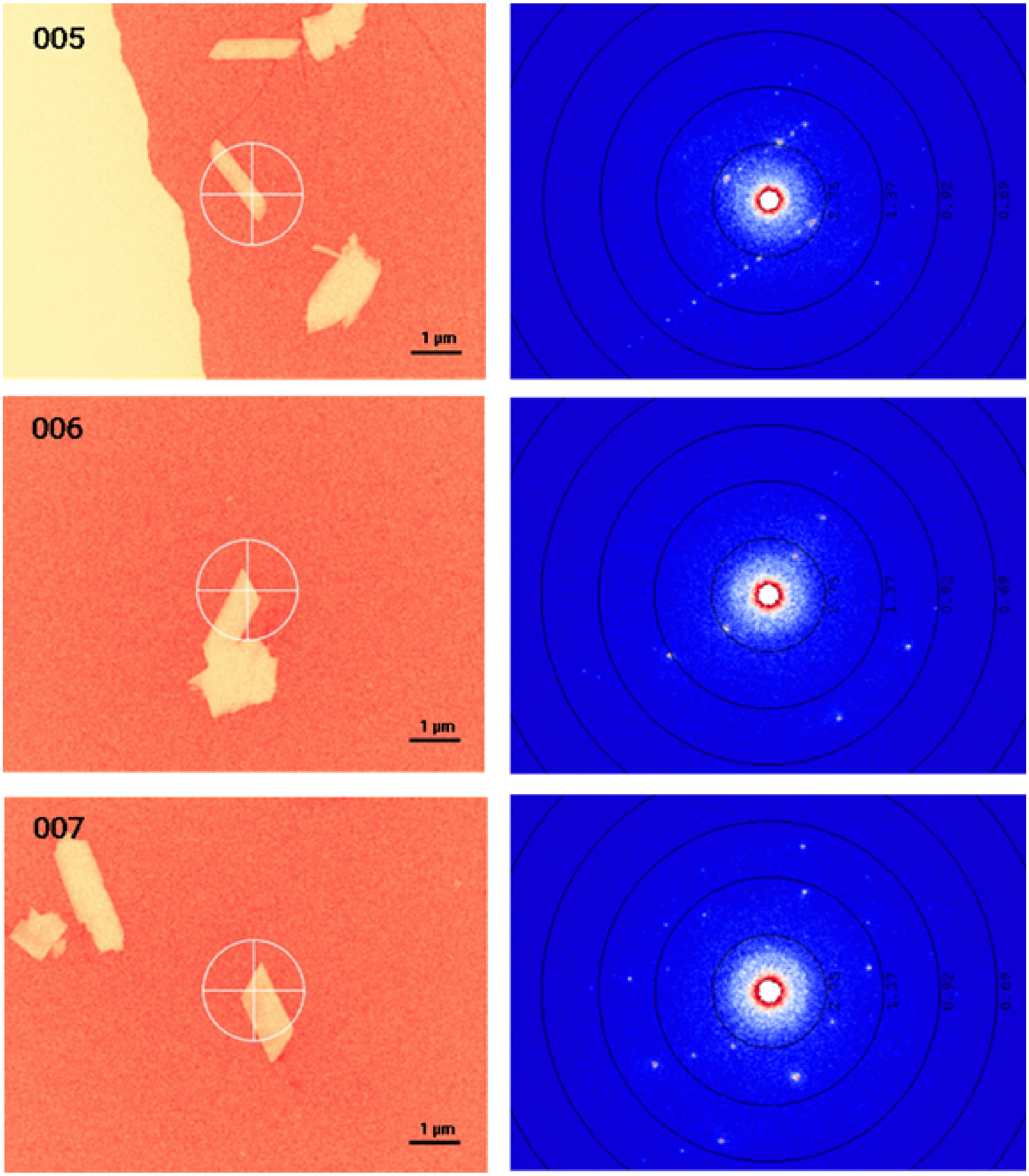

Figure S14: **Images from 3D ED experiments.** Grain snapshots and diffraction images for all selected 3D ED measurements. The colorless plate-like crystals are ca. 100 nm thick.

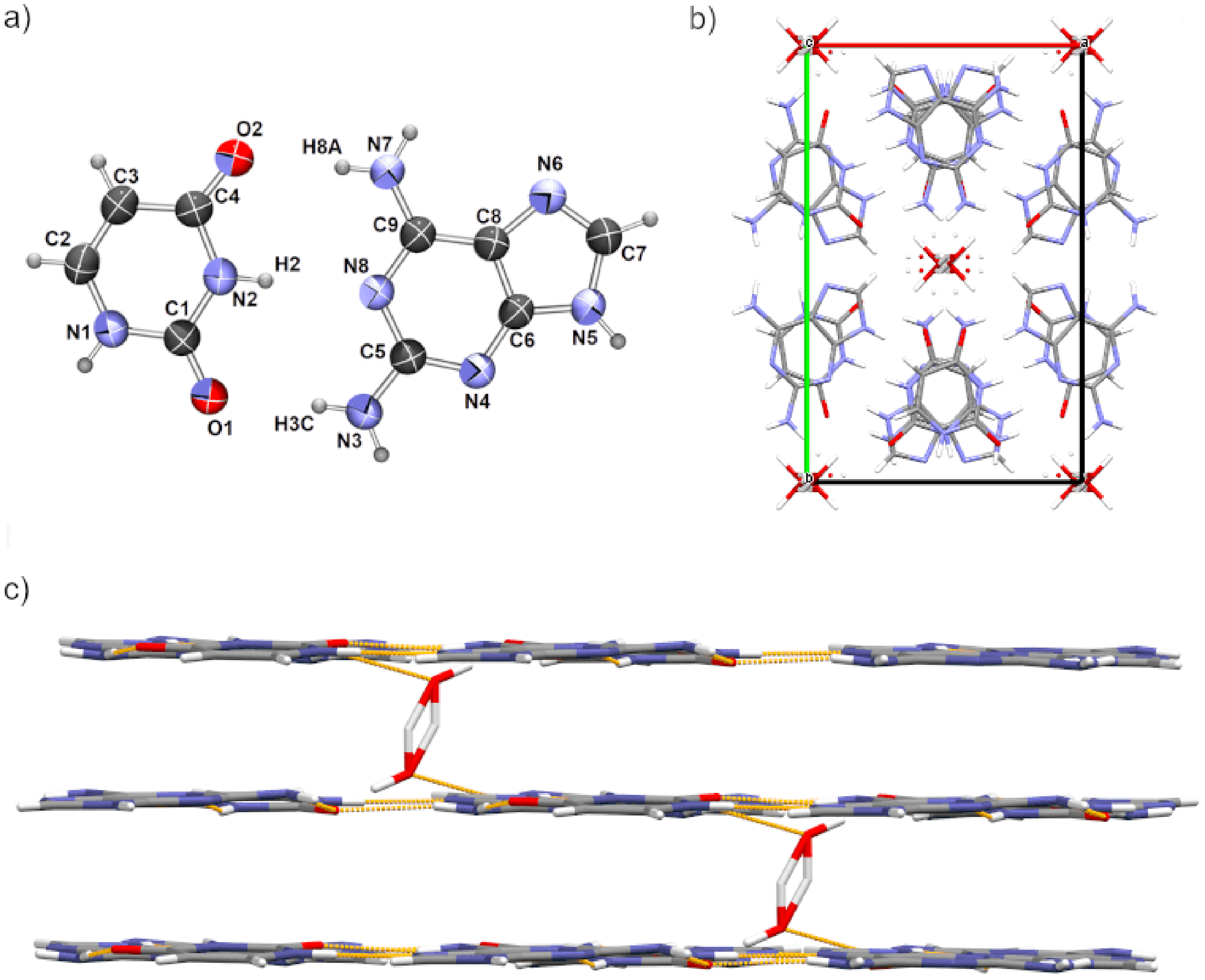

Figure S15: **D-U hyd crystal structure.** a) ORTEP-3 drawing of asymmetric unit of **D-U hyd** with atom numbering scheme. Displacement ellipsoids are drawn for the probability of 50% and hydrogen atoms are shown as spheres of arbitrary radii. Disordered water molecule has been omitted. b) Disordered water molecules lie in the channels parallel to the crystallographic *c*-axis. c) Disordered water molecules form inter-layer hydrogen bonds.

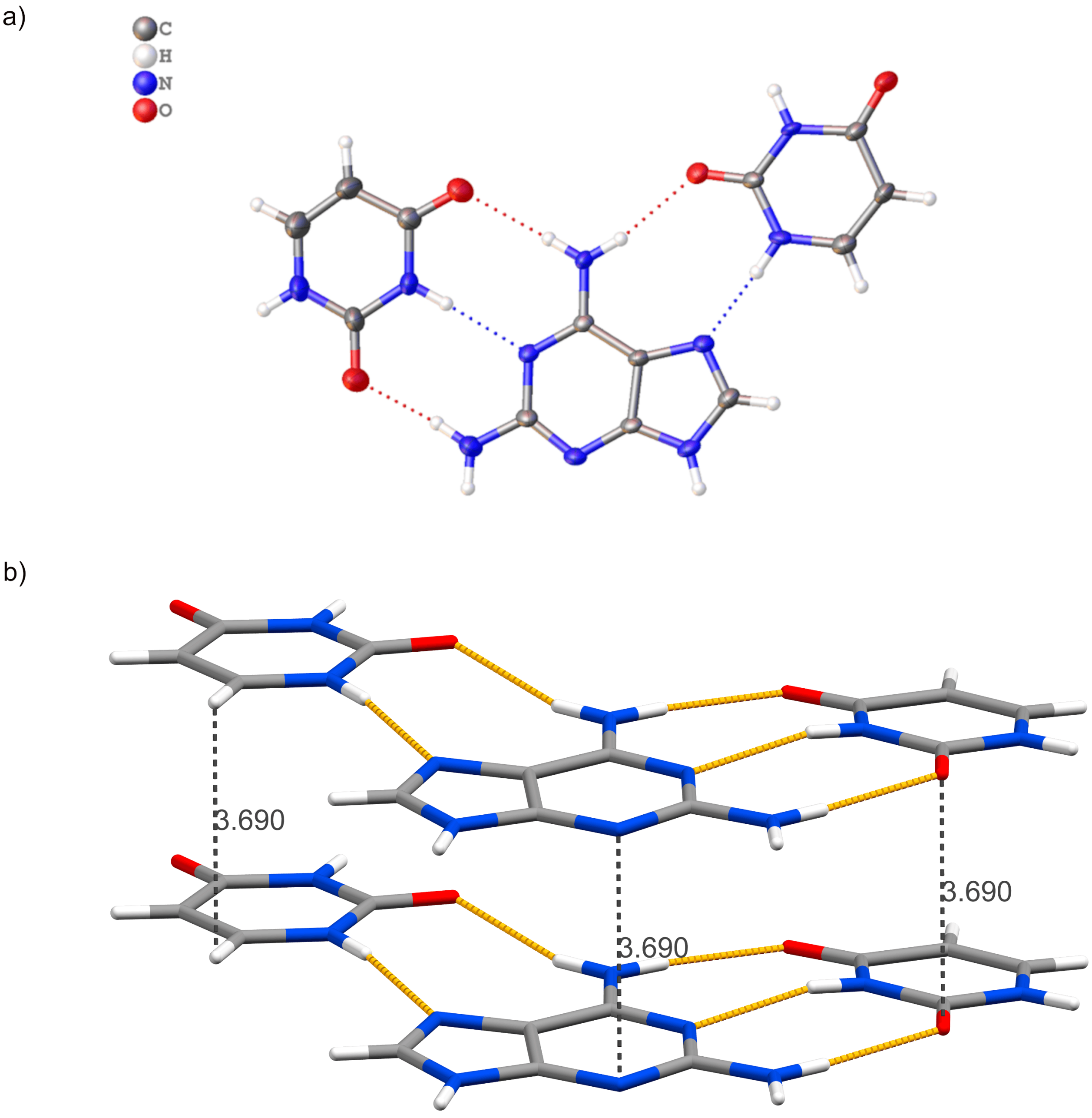

Figure S16: **D-U anhyd crystal structure.** a) Displacement ellipsoid plot of **D-U anhyd**. Displacement ellipsoids are drawn at the 50% probability level. Red and blue dotted lines represent N–H$\cdots$O and N–H$\cdots$H hydrogen bonding, respectively. b) D-U hydrogen bonding and homomeric parallel displaced π-π stacking interactions in **D-U anhyd**.

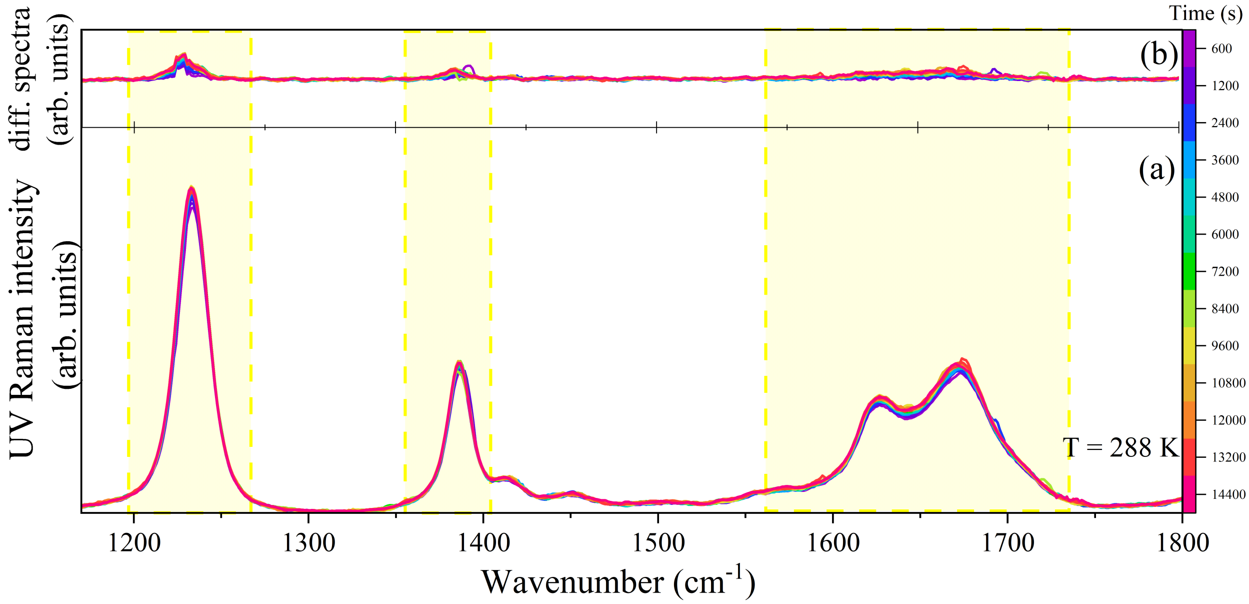

Figure S17: **UVRR measurements on U solutions upon continuous irradiation.** Effect of UV irradiation directly measured via UV resonance Raman spectroscopy at 266 nm for U aqueous solutions, collecting one spectrum each 10' of exposure time (time is traced by the color scale on the right side). Spectra reported in a) Raman intensities, b) as a difference with respect to the spectrum at time 0. More details provided in Tables S3 and S4.

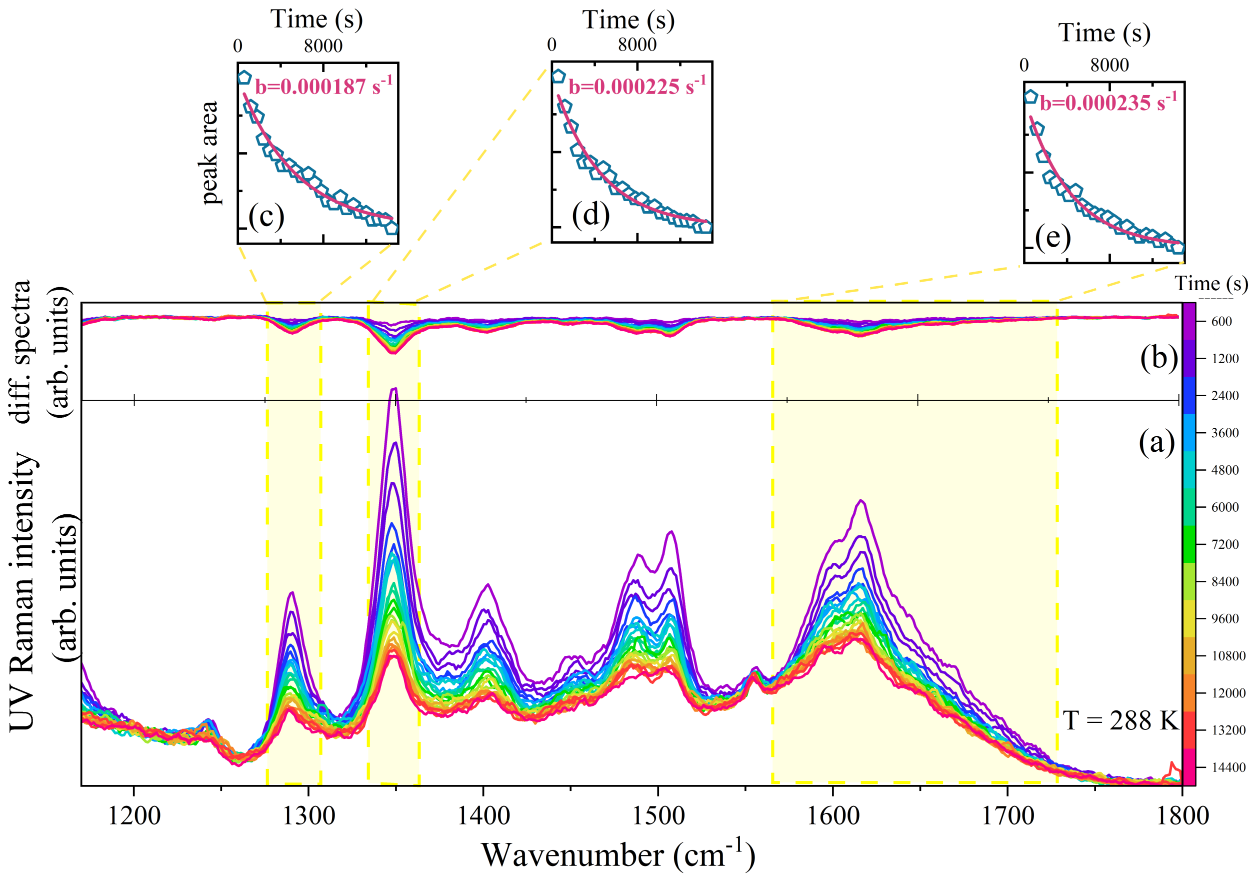

Figure S18: **UVRR measurements on D solutions upon continuous irradiation.** Effect of UV irradiation directly measured via resonance Raman spectroscopy at 266 nm for D aqueous solutions, collecting one spectrum each 10' of exposure time (time is traced by the color scale on the right side). Spectra reported in a) Raman intensities, b) as a difference with respect to the spectrum at time 0. Upper insets (c-e): time traces and monoexponential fitting coefficient of spectral regions highlighted in yellow. More details provided in Tables S3 and S4.

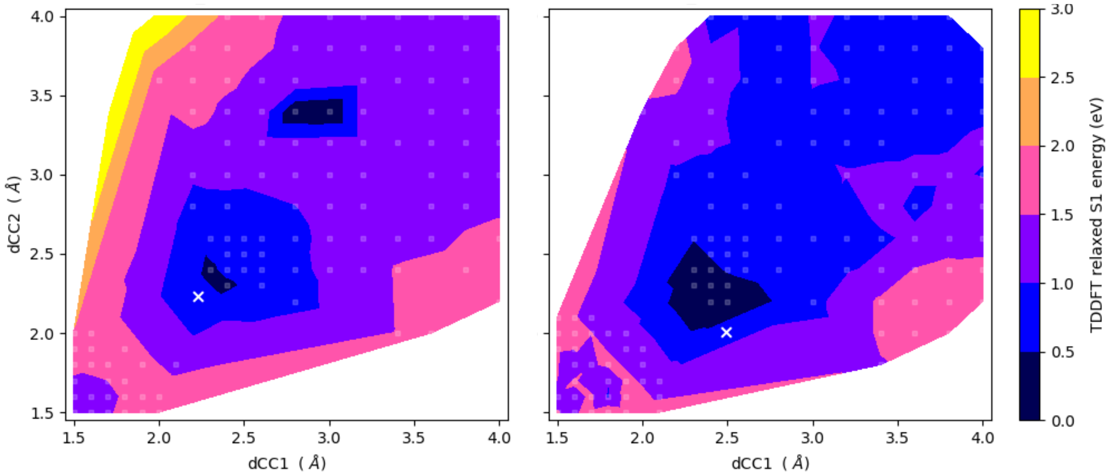

Figure S19: **TDDFT relaxed PES of S1 for UU and DUU.** Relaxed first excited state ωB97XD DFT PES inferred from the points that successfully converged (marked in shaded grey). The minima for UU potential in the region of dCC1, dCC2 = $\sim$(3.0,3.5) corresponds to a transfer of a proton to the oxygen of the nearby U molecule (Figure S22). The region where the CI were found are marked with a white cross (see also Fig. 5).

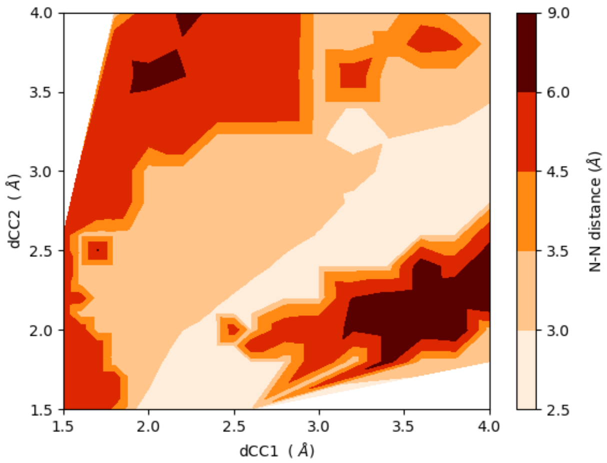

Figure S20: **H-bonded N(D)-N(U) distance.** Distance of the nearby N atom on D acting as a donor of hydrogen bond and its acceptor on U, computed on the ground state potential for DUU.

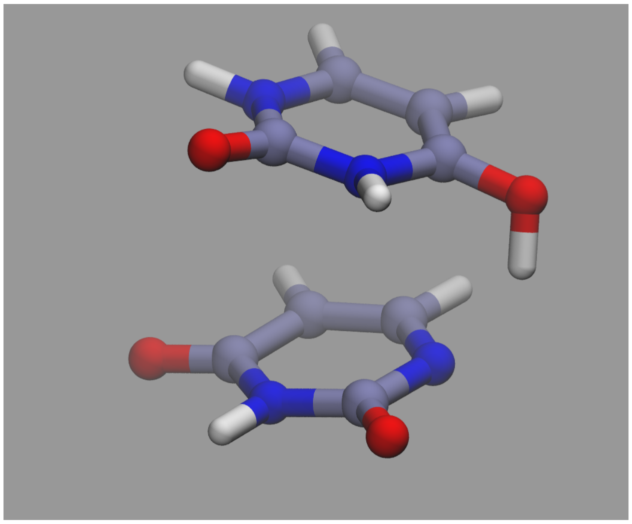

Figure S21: **Unexpected S_1 relaxed UU structure showing proton transfer.** Structure corresponding to the basin seen for UU first excited state relaxation at ωB97XD TDDFT and corresponding to a proton transfer. This structure correspond to dCC1=3.0 Å and dCC2=3.4Å. An attempt to fully minimize this configuration did not terminate successfully.

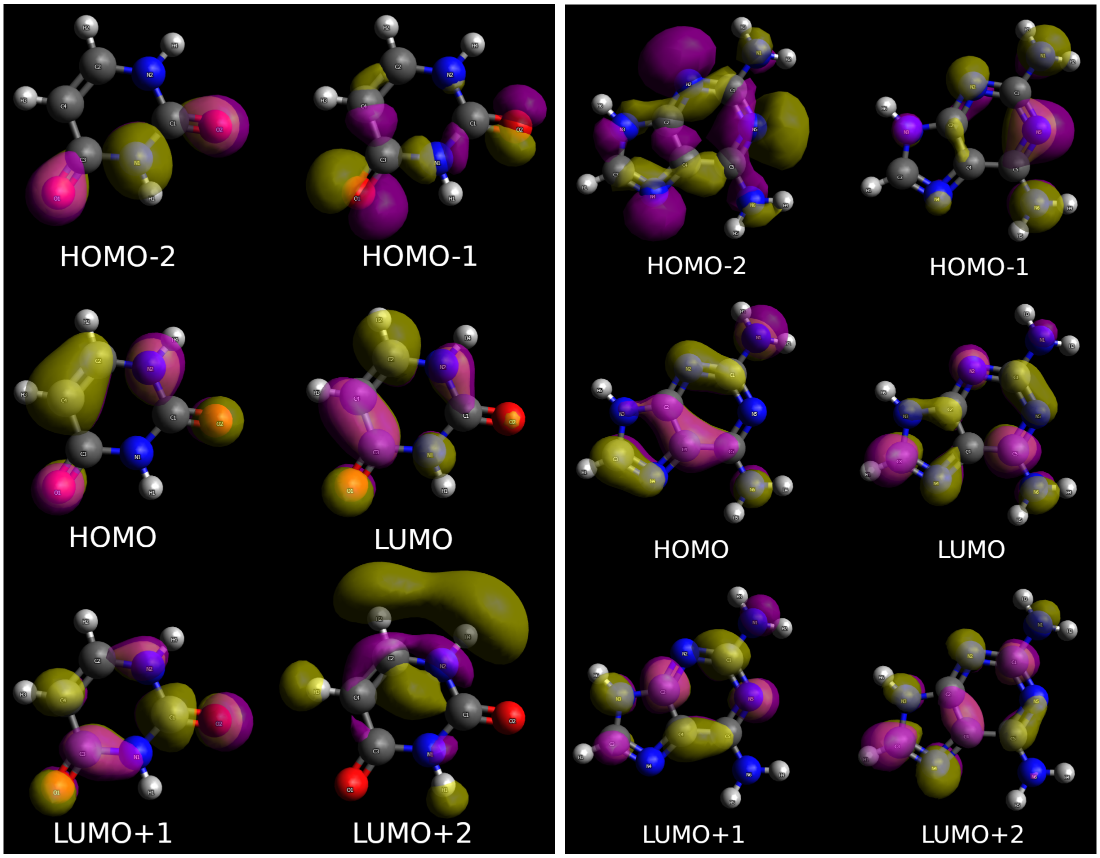

Figure S22: **MOs for isolated D and U.** Molecular orbitals for U (left) and D (right).

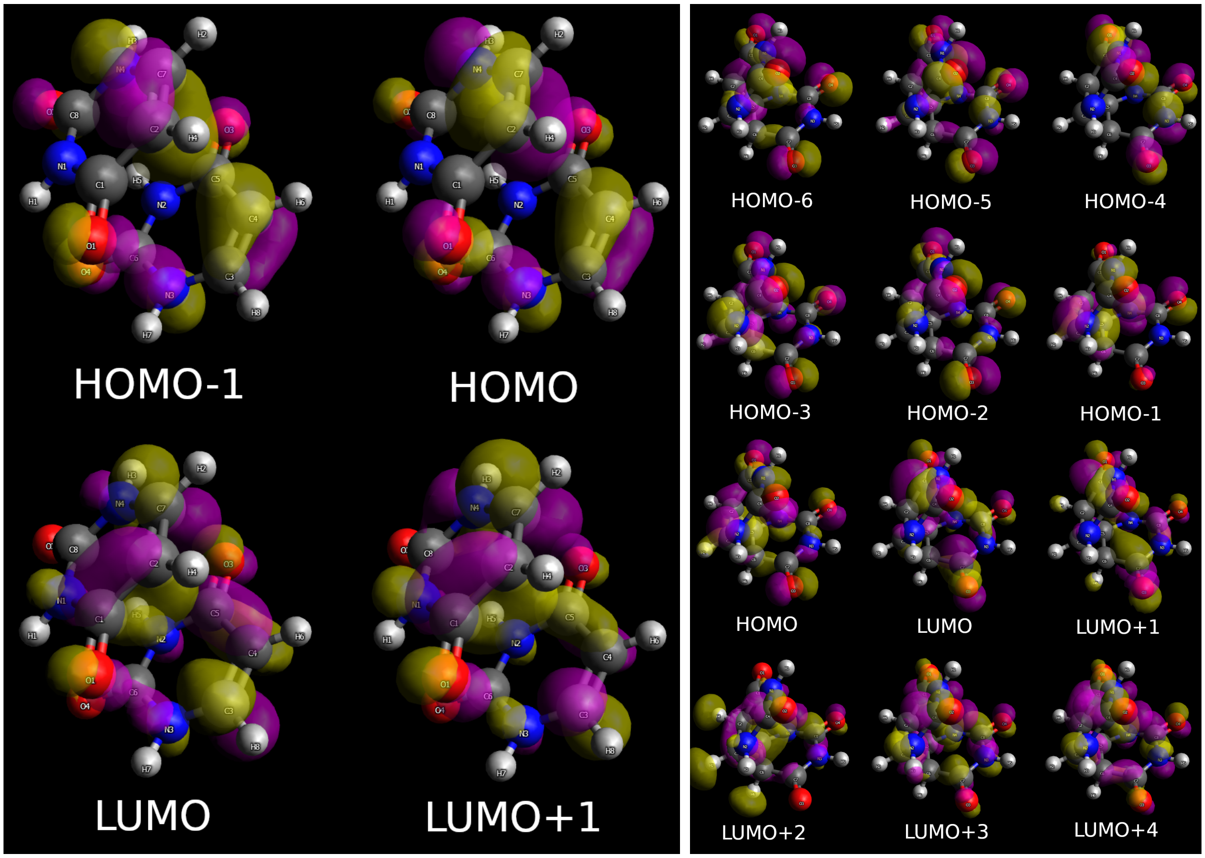

Figure S23: **MOs for UU structures.** Molecular orbitals for U(π)U (left) and U□U (right).

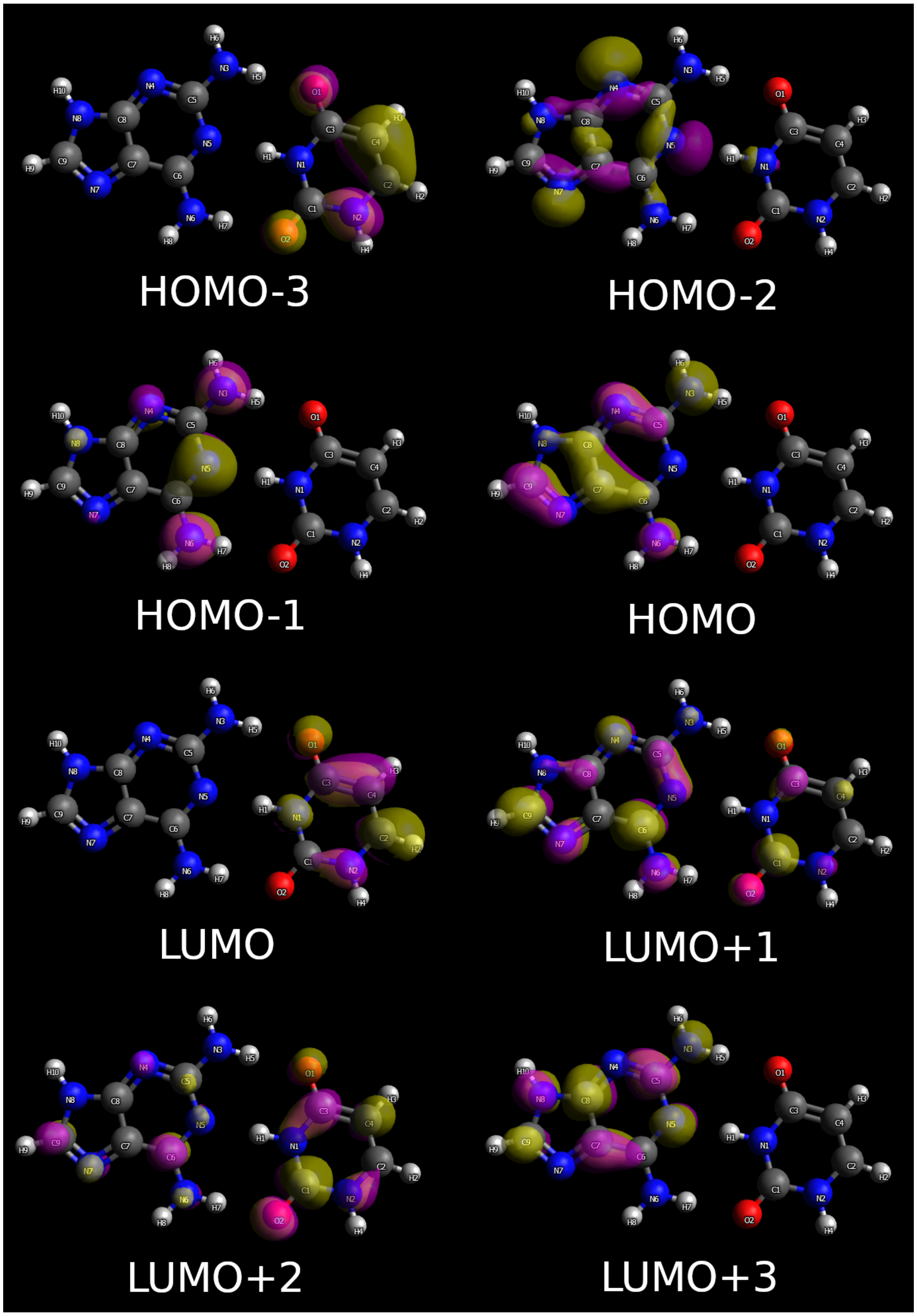

Figure S24: **MOs for DUU structures.** Molecular orbitals for D:U.

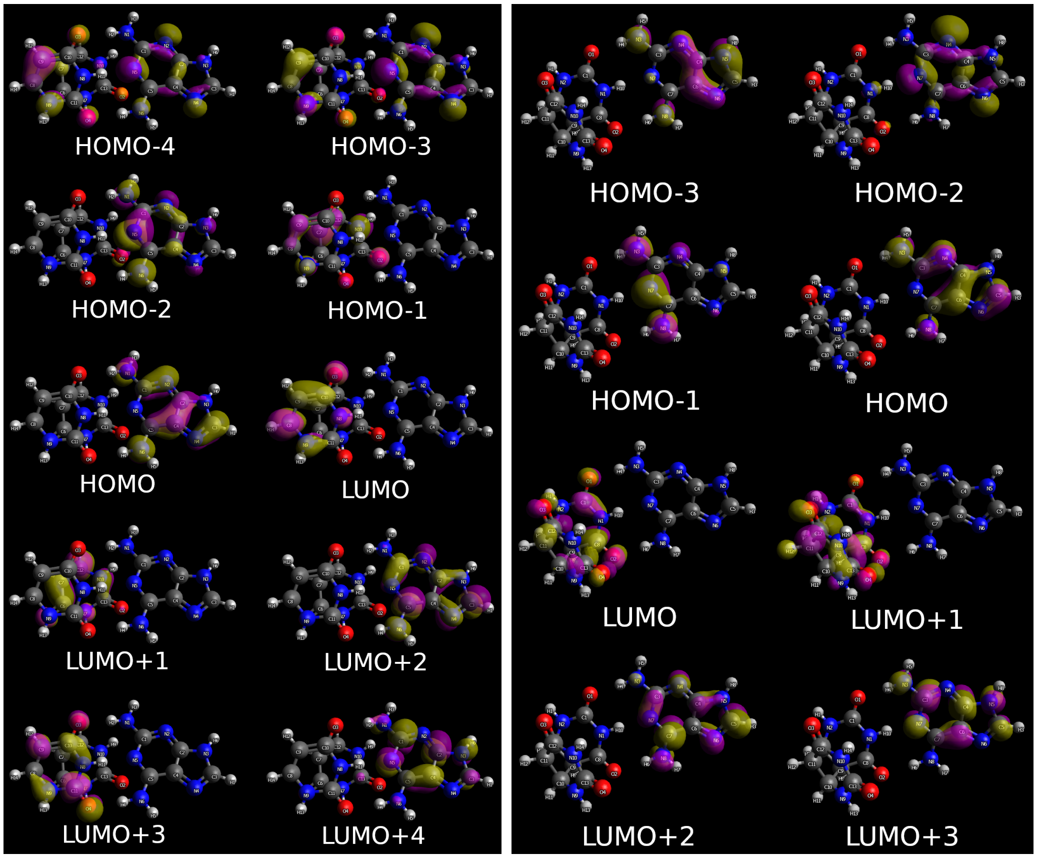

Figure S25: **MOs for DUU structures.** Molecular orbitals for D:U(π)U (left) and D:U□U (right).

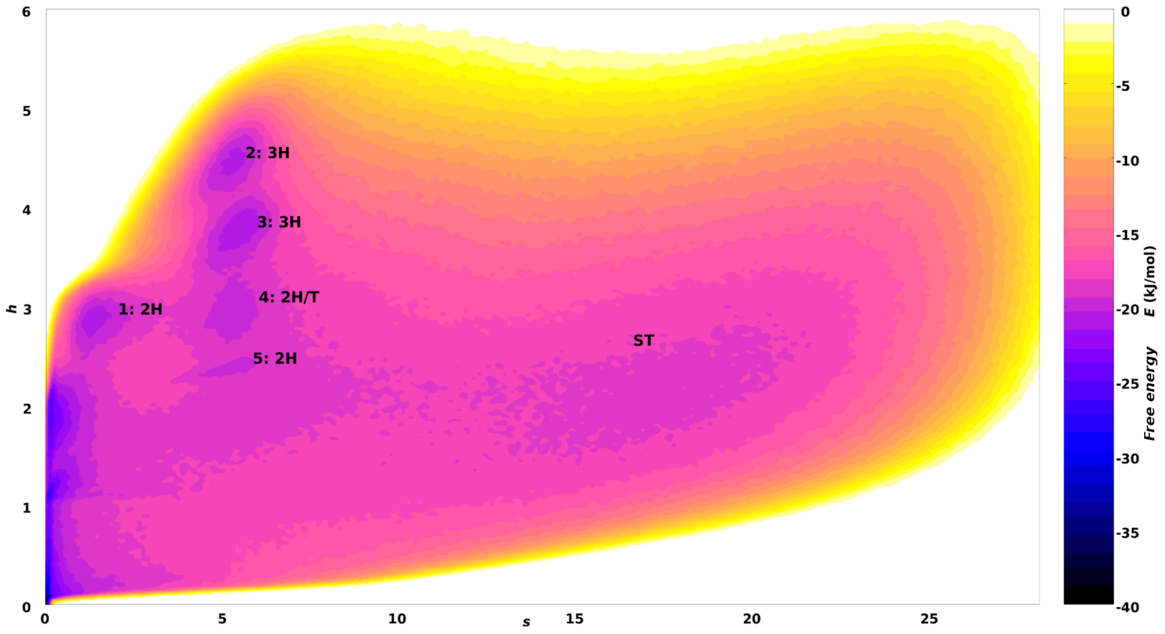

Figure S26: **Free energy surface of U and D in water - preliminary results.** The system, containing containing one uracil and one 2,6-diaminopurine molecules (1u:1d) solvated with water (TIP3P model), was simulated using well-tempered metadynamics in combination with reparameterized AMBER99 force-field. The hills were added every 250 steps, tau was set to 100, while bias factor was 10. The box size was 50 x 50 x 50 Å^3. The system was minimized and equilibrated (1. NVT (velocity-rescale thermostat, 298.15K, 0.25 ns), 2. NpT (Berendsen barostat, 1 bar, 0.25 ns). The equations of motion were integrated using a 0.5 fs time step and `md` algorithm. Long-range electrostatic interactions were treated using the Particle Mesh Ewald (PME) approach, with a fourth-order cubic interpolation, grid size of 0.12 nm and tolerance of 10^{-5}. Neighbour lists were updated every 5 steps. A cutoff of 10 Å was used for van der Waals interactions, real space Coulomb interactions, and updating neighbour lists. The collective variables for biasing were defined as coordination numbers (i.e. number of contact modulated by switching function) describing two non-covalent interactions: (pi-)pi stacking s and hydrogen bonds h. The production run was done as NpT, for a total of 800 ns. The preliminary free energy surface as obtained by summing added hills at this stage of the simulation, is shown in Figure SN1 in terms of the two collective variables s and h. Several minima basins can be identified. The wide basin on the right, described with high s, corresponds to the stacking configuration of two nucleobases. Other minima basins are related to hydrogen-bonding and T-shaped/H-bonding configurations as shown in Table S9. The work is in progress and it will be subject of a future publication.

Table S1: **Analysis of interactions from crystal structures.** List of interaction energies V_{AB} computed by performing DFT geometry relaxation for a set of di-molecular and multi-molecular complexes relevant for the crystal structure of **D-U hyd** and **DU anhyd**. Note: a minimum was not found for **UU-pi** dimer which rendered a flat hydrogen-bonded structure (^{a}) or a T-shaped configuration (^{b}) instead, therefore the values reported here correspond to a plateau in the potential energy corresponding to a π-stacking dimer structure.

	cluster description	N hydr. bonds	V_{AB} (kcal/mol)
D-U hyd	D : U (W-C, water)	4	-25.1
	D : U (W-C)	3	-18.5
	D : D (HB intralayer)	2	-18.0
	D : U (HB intralayer)	2	-16.6
	D : water : U (interlayer, water bridged)	2	-11.8
	D : water : U (intralayer, water bridged)	2	-10.2
	D U (π stacking)	0	-9.6
	D D (π stacking)	0	-7.5
	U U (π stacking)	0	-6.8 ^{a}
D-U anhyd	D : U_3 (intralayer)	7	-54.9
	D : U (HB intralayer)	3	-18.5
	D : U (HB intralayer)	3	-20.5
	D : U (HB intralayer)	2	-16.6
	D D (π stacking)	0	-7.2
	U U (π stacking)	0	-2.8 ^{b}

Table S2: **Crystal formation energies.** Detailed formation energies according to the procedure described earlier in the supporting information.

(kcal/mol)	DU hyd	DU anhyd
$E^{\text{form}}_{\text{rigid cell}}$	-7.10	2.20
$E^{\text{form}}_{\text{full}}$	-8.53	-3.31

Table S3: **UVRR peak assignements.** Assignments of main resonance Raman peaks and reference in the literature, [a]: for D at 257 nm,[1] for U at 266 nm[2] (and Ref. 24 therein).

molecule	mode[a]	this work	literature[a]	note
U	C4C5, N1C2	$\sim$ 783	789	cuvette interference
D	C4N9	1159	1158	weak
U	C5H, C6H	1234	1235	
D	N7C5, N7C8H, N9C8	1291	1291	weak
D	N1C6	1348	1348	
U	C2N3, N3H	1387	1388	
D	C8N7, N9C8	1508	1510	doublet, weak
D	N3C2, C2N1	1616	1613	broad
U	C5C6	1626	1623	
U	C4O	1672	1664	

Table S4: **UVRR decay fit details and parameters.** UVRR scattering was measured each 10' at the temperature of 288 K. The fit was obtained by fitting the normalized integral time series (i.e. setting largest value to 1 and smallest to 0) on the defined range $I(t)$ with a mono-exponential single-parameter decay time profile: $I(t) \rightarrow y(t) = -\exp(bt)$. The table report the value and the estimated standard deviation of the fit parameter b.

system	range (cm^{-1})	b (s^{-1})	σ_b (s^{-1})
D-U	1203. $\div$ 1262.5	2.912e−04	9.3e−06
	1275 $\div$ 1310	2.344e−04	8.5e−06
	1325.5 $\div$ 1365	2.706e−04	9.5e−06
	1366 $\div$ 1404	2.938e−04	9.9e−06
	1561 $\div$ 1730	3.443e−04	1.65e−05
D	1203. $\div$ 1262.5	1.871−04	5.2e−06
	1325.5 $\div$ 1365	2.248−04	7.2e−06
	1561 $\div$ 1730	2.351e−04	8.2e−06

Table S5: **In-vacuum DFT energies and geometry information.** Details for DFT ground-state minima for UU and D:UU (and its molecular isolated components) obtained at ωB97XD level of theory. For comparison, the CC distances of the structures identified as conical intersections via CASSCF are dCC1 = 2.49 and dCC2 = 2.01 Å for D:UU(CI); dCC1 = 2.23 and dCC2 = 2.23 Å for UU(CI). [a] when available, relative energy respect to the isolated molecular fragments, with Basis Set Superposition Error (BSSE) correction;[3] [b] BSSE-corrected energies[3] and geometries.

	E_{DFT} (Ha)<br>(E^{a}_{rel}) (eV)	dCC(Å)<br>dCC1<br>dCC2	dHB(Å)<br>dC2(U)N(D)<br>dN3(U)N(D)<br>dC4(U)N(D)
U(π)U [b]	-829.381202<br>(-0.3567)	3.77<br>3.79	
U□U	-829.383019<br>(-0.1544)	1.57<br>1.57	
D:U(π)U [b]	-1351.945263<br>(-1.3911)	3.77<br>3.65	2.97<br>2.90<br>2.98
D:U□U	-1351.93747151<br>(-0.9281)	1.58<br>1.58	2.96<br>2.93<br>2.96
U	-414.684046093		
D	-522.519501332		
D:U [b]	-937.232667<br>(-0.7924)		2.94<br>2.92<br>2.97

Table S6: **TDDFT transitions: energies and oscillator strenghts.** Details for TD-DFT calculations for all ground-state minima for UU and D:UU (and its molecular isolated components) obtained at [w]) ωB97XD level of theory and [c]) CAM-B3LYP : energies and oscillator strenghts for first excited states. [b] evaluated at BSSE-corrected geometries.

	$S_0 \rightarrow S_1$ E (eV) (f_{osc})	$S_0 \rightarrow S_2$ E (eV) (f_{osc})	$S_0 \rightarrow S_3$ E (eV) (f_{osc})	$S_0 \rightarrow S_4$ E (eV) (f_{osc})
^{b} U(π)U ^{w}	5.109 (0.0001)	5.115 (0.0002)	5.374 (0.017)	5.630 (0.235)
^{b} U(π)U ^{c}	5.107 (0.0001)	5.113 (0.0002)	5.377 (0.016)	5.623 (0.239)
U□U ^{w}	5.237 (0.0003)	5.272 (0.0014)	6.203 (0.003)	6.214 (0.002)
U□U ^{c}	5.202 (0.0003)	5.261 (0.0014)	6.203 (0.002)	6.206 (0.002)
^{b} D:U(π)U ^{w}	5.182 (0.0001)	5.262 (0.122)	5.295 (0.003)	5.360 (0.036)
^{b} D:U(π)U ^{c}	5.187 (0.0002)	5.266 (0.108)	5.294 (0.015)	5.387 (0.039)
^{b} D:U□U ^{w}	5.089 (0.160)	5.232 (0.0008)	5.316 (0.002)	5.341 (0.002)
^{b} D:U□U ^{c}	5.032 (0.042)	5.122 (0.122)	5.225 (0.001)	5.318 (0.002)
U ^{w}	5.090 (0.0001)	5.632 (0.170)		
U ^{c}	5.093 (0.0001)	5.638 (0.173)		
D ^{w}	5.1878 (0.151)	5.693 (0.019)	5.724 (0.169)	
D ^{c}	5.198 (0.152)	5.709 (0.160)	5.739 (0.027)	
^{b} D:U ^{w}	5.097 (0.148)	5.212 (0.002)	5.390 (0.019)	5.617 (0.159)
^{b} D:U ^{c}	5.092 (0.01)	5.209 (0.149)	5.361 (0.003)	5.614 (0.183)

Table S7: **Contribution to each TDDFT transition.** Details for TDDFT calculations for all ground-state minima for UU and D:UU (and its molecular isolated components) obtained at ωB97XD level of theory. Besides excitation energies and oscillator strengths, the composition in term of configuration coefficients (only when $|c| > 0.15$) for each transition are shown. [b] evaluated at BSSE-corrected geometries.

	$S_0 \rightarrow S_1$	$S_0 \rightarrow S_2$	$S_0 \rightarrow S_3$	$S_0 \rightarrow S_4$
	composition (MO coeff.)			
[b] U(π)U	H-3→L(0.16)<br>H-3→L+1(0.22)<br>H-2→L(0.42)<br>H-2→L+1(0.38)	H-3→L(0.46)<br>H-3→L+1(-0.34)<br>H-2→L+1(0.21)	H-1→L+1(0.29)<br>H→L(0.63)<br>H-2→L(-0.18)	H-1→L(0.41)<br>H→L+1(0.53)
U□U	H-6→L(0.17)<br>H-5→L+1(0.16)<br>H-3→L(0.30)<br>H-2→L+1(0.37)<br>H→L(0.41)	H-6→L+1(0.18)<br>H-5→L(0.18)<br>H-3→L+1(0.27)<br>H-2→L(0.44)<br>H→L+1(0.33)	H-6→L(0.39)<br>H-5→L+1(0.27)<br>H→L(-0.37)	
[b] D:U(π)U	H-9→L+1(0.20)<br>H-5→L+1(0.58)<br>H-5→L+5(0.17)<br>H-3→L+1(0.18)	H→L+2(0.57)<br>H→L+4(-0.32)	H-8→L(0.49)<br>H-7→L(0.39)	H-3→L(-0.27)<br>H-1→L(-0.33)<br>H-1→L+1(0.48)
[b] D:U□U	H→L+2(0.62)<br>H→L+3(-0.24)	H-4→L(0.31)<br>H-4→L+1(-0.26)<br>H-6→L(0.26)<br>H-6→L+1(-0.35)<br>H-12→L+1(0.18)	H→L(0.41)<br>H-4→L(0.15)<br>H-7→L(0.18)<br>H-8→L(0.18)<br>H-9→L(-0.28)<br>H-9→L+1(-0.15)<br>H-13→L(0.16)	H→L(0.51)<br>H→L+1(0.17)<br>H-9→L(0.23)
U	H-3→L(0.17)<br>H-2→L(0.64)<br>H-2→L+1(0.19)	H→L(0.68)		
D	H→L(0.63)<br>H→L+1(-0.23)	H-2→L(0.58)<br>H-1→L(-0.28)<br>H→L+1(-0.20)	H-2→L(0.30)<br>H→L(0.27)<br>H→L+1(0.54)<br>H-1→L(0.17)	
[b] D:U	H→L+1(0.59)<br>H→L+2(-0.20)<br>H→L+3(-0.25)	H-6→L(0.18)<br>H-5→L(0.58)<br>H→L(-0.20)<br>H-5→L+2(0.16)	H→L(0.65)<br>H-5→L(0.19)	H-3→L(0.63)<br>H→L+2(0.23)

Table S8: **Details for CASSCF calculations for identifying the conical intersections.** Compositions, roots and main contributions ($|c|$ >0.10) . To speed up the calculations, the actual input for these calculations were taken from the DFT calculations of the scan with small TDDFT gaps. Both the optimised CI structures obtained as well as the input structures for CASSCF CI optimisations are reported at the end of these Supplementary Information).

UU (4,4)	composition: H-1, H, L, L+1
ROOT 1	EIGENV. −824.8339660
	1100 (0.945), 1001 (-0.173), 1ab0 (0.167), 0110 (-0.164), 1010 (0.101)
ROOT 2	EIGENV. −824.8339240 (+ 0.0011 eV)
	1ab0 (0.936), abab (0.265), 1100 (-0.166), 0ab1 (-0.116)
D:UU (6,6)	composition: H-4, H-3, H-1, L, L+1, L+3
ROOT 1	EIGENV. −1344.434957
	11ab00 (0.815), 111000 (-0.507), a1ba0b (0.190)
ROOT 2	EIGENV. −1344.434942 (+ 0.0004 eV)
	111000 (0.820), 11ab00 (0.497) , 110001 (-0.109), 011100 (-0.106)

Table S9: **Classification of free-energy minima of D and U..** From the free energy reported Figure 26, Several minima basins can be identified. The wide basin, described with large s, corresponds to the stacking configuration of two nucleobases. Other minima basins are related to hydrogen-bonding and T-shaped/H-bonding configurations as shown in Table 1. Even though the deepest minima is (0,0) and it is thermodynamically most favourable, from these preliminary results we can say that hydrogen-bonded between uracil and 2,6-diaminopurine are possible even in aqueous solutions and these can be classified as minimum-energy configurations. The relevance of these is expected to be larger especially at large concentrations. The 2: 3H corresponds to a configuration where uracil and 2,6-diaminopurine interact with 3 hydrogen bonds, the same included in the model system used for DFT (Fig. 5) and also encountered in the crystal structure. The work is in progress and it will be subject of a future publication.

abbreviation	*ΔE* (kJ/mol)	*structure*
**0**	0.00	Nucleobases do not interact with each other, but solely with water
**1:** 2H	17 ± 5	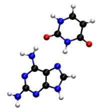
**2:** 3H	15 ± 5	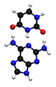
**3:** 3H	14 ± 5	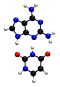
**4:** 2H/T	16 ± 5	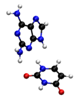
**5:** 2H	16 ± 5	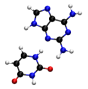
**6:** ST	17 ± 5	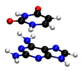

Coordinates in cartesian format The following contains the list of coordinates for all the structures that were obtained from the DFT simulation in vacuum and characterised in this work.

U(π)U

```
24
UU_pi_dimer_wB97XD_gs #relevant for TD-DFT: Fig 5, Tab.S6
N      1.2288670000   -1.1533550000    0.5884430000
C      0.9594670000   -1.5318980000   -0.7363650000
C      1.4939000000   -0.6074050000   -1.7278220000
C     -2.2209110000   -0.1671550000   -1.2871930000
C     -1.6444820000    1.0408150000   -1.4242340000
C     -0.9842560000    1.6587700000   -0.2817480000
N     -1.1357360000    0.9382260000    0.9131950000
C     -1.6728760000   -0.3266140000    1.0711260000
N     -2.2657650000   -0.8172290000   -0.0810280000
C      2.0736980000    0.5384990000   -1.3268710000
N      2.2396050000    0.8418960000   -0.0001550000
C      1.7624630000    0.0445340000    1.0275450000
O      0.3090600000   -2.5338750000   -0.9716160000
O      1.7998540000    0.3821200000    2.1924540000
H      0.8021090000   -1.7197310000    1.3139500000
H      2.4362060000    1.2875360000   -2.0204850000
H      2.5136220000    1.7723000000    0.2803720000
H      1.3578300000   -0.8435760000   -2.7734760000
O     -0.3254120000    2.6827210000   -0.3050410000
O     -1.6221460000   -0.9589250000    2.1051950000
H     -0.6362290000    1.2927810000    1.7220950000
H     -1.6047390000    1.5510200000   -2.3759560000
H     -2.5525830000   -1.7838560000   -0.0297350000
H     -2.6778590000   -0.6968490000   -2.1145040000
```

```
24
UU_pi_dimer_wB97XD_gs_BSSE #BSSE-corrected
N      1.4558490000   -0.8678590000    0.7101700000
C      1.3305650000   -1.4656200000   -0.5538140000
C      1.6517580000   -0.5688720000   -1.6573430000
C     -2.1202670000   -0.5983000000   -1.3151760000
C     -1.8281340000    0.7026380000   -1.4930560000
C     -1.3514840000    1.4964960000   -0.3672840000
N     -1.3432610000    0.7973900000    0.8494990000
C     -1.6076900000   -0.5436270000    1.0581310000
N     -2.0324900000   -1.1998900000   -0.0867380000
C      1.9396790000    0.7209320000   -1.4071140000
N      1.9926560000    1.2172550000   -0.1305250000
C      1.7073060000    0.4610530000    0.9957220000
O      0.9645080000   -2.6212330000   -0.6573740000
O      1.6724270000    0.9333780000    2.1121650000
H      1.1805570000   -1.4334920000    1.5051870000
H      2.1422290000    1.4418530000   -2.1902730000
H      2.0719710000    2.2101350000    0.0340770000
H      1.6080930000   -0.9612870000   -2.6633100000
O     -0.9643560000    2.6489740000   -0.4198430000
O     -1.4728330000   -1.1033060000    2.1250380000
H     -0.9706100000    1.2914860000    1.6528740000
H     -1.8964840000    1.1774430000   -2.4613440000
H     -2.1361500000   -2.1993080000    0.0098740000
H     -2.4372440000   -1.2458090000   -2.1242030000
```

U□U (Uracil CPD)

```
24
UU_dimer_wB97XD_gs #relevant for TD-DFT: Fig 5, Tab.S6
N      1.2139230000    1.3220300000   -0.8477770000
C      0.3789370000    1.8499950000    0.1079810000
C      0.2699000000    1.0680120000    1.3930050000
C      1.0680420000   -0.2496840000    1.4858740000
N      2.0630910000   -0.4267870000    0.4632620000
C      2.0649530000    0.2088830000   -0.7504660000
O     -0.2699000000    2.8573180000   -0.0804960000
O      2.7653060000   -0.1157510000   -1.6830940000
H      1.2680420000    1.8147260000   -1.7307970000
H      1.5413740000   -0.3824540000    2.4604050000
H      2.5513410000   -1.3120330000    0.4363740000
H      0.4247920000    1.7677000000    2.2159810000
C     -1.0680420000    0.2496840000    1.4858740000
C     -0.2699000000   -1.0680120000    1.3930050000
C     -0.3789370000   -1.8499950000    0.1079810000
N     -1.2139230000   -1.3220300000   -0.8477770000
C     -2.0649530000   -0.2088830000   -0.7504660000
N     -2.0630910000    0.4267870000    0.4632620000
O      0.2699000000   -2.8573180000   -0.0804960000
O     -2.7653060000    0.1157510000   -1.6830940000
H     -1.2680420000   -1.8147260000   -1.7307970000
H     -0.4247920000   -1.7677000000    2.2159810000
H     -2.5513410000    1.3120330000    0.4363740000
H     -1.5413740000    0.3824540000    2.4604050000
```

```
24
UU_dimer_wB97XD_gs_BSSE #BSSE-corrected: Tab.S5
N      1.6749970000    0.7772550000   -0.8239620000
C      1.1840680000    1.5727560000    0.1841270000
C      0.7051900000    0.8482130000    1.4163920000
C      0.8615280000   -0.6853580000    1.4558250000
N      1.6284520000   -1.2368060000    0.3707020000
C      1.9200740000   -0.6050750000   -0.8070060000
O      1.0865330000    2.7763840000    0.0720040000
O      2.3886190000   -1.1616370000   -1.7756540000
H      1.9542210000    1.2419310000   -1.6790980000
H      1.2789230000   -1.0477890000    2.3967260000
H      1.6972400000   -2.2430510000    0.3043460000
H      1.1090970000    1.3712210000    2.2838730000
C     -0.8615160000    0.6853540000    1.4558300000
C     -0.7051790000   -0.8482170000    1.4163900000
C     -1.1840590000   -1.5727550000    0.1841230000
N     -1.6749980000   -0.7772510000   -0.8239600000
C     -1.9200590000    0.6050810000   -0.8070030000
N     -1.6284410000    1.2368060000    0.3707090000
O     -1.0865560000   -2.7763870000    0.0720090000
O     -2.3886410000    1.1616410000   -1.7756360000
H     -1.9542430000   -1.2419270000   -1.6790890000
H     -1.1090850000   -1.3712270000    2.2838700000
H     -1.6972360000    2.2430510000    0.3043600000
H     -1.2789110000    1.0477790000    2.3967330000
```

D:U(π)U

```
41
 DUU_pi_dimer_wB97XD_gs #relevant for TDDFT: Fig 5, Tab.S6
N     -1.0769630000    2.4631920000   -0.2045150000
N     -3.2514480000    1.7087690000    0.0156300000
N     -5.3528430000    0.5027960000    0.2775470000
N     -4.6604420000   -1.5826120000   -0.1653180000
N     -1.4571270000    0.2206960000   -0.6032880000
N     -1.7277820000   -2.0713400000   -0.8610100000
C     -1.9886040000    1.4222550000   -0.2884520000
C     -4.0145800000    0.6093530000    0.0052540000
C     -5.6753920000   -0.8337200000    0.1604040000
H     -6.6836650000   -1.1863110000    0.3296050000
C     -3.6104930000   -0.6893860000   -0.2696270000
C     -2.2459140000   -0.8671890000   -0.5746840000
H     -0.2786470000    2.3913030000   -0.8393360000
H     -1.5272170000    3.3690380000   -0.2145300000
H     -0.7183940000   -2.2037390000   -0.8148050000
H     -2.3156100000   -2.8719770000   -0.6854680000
H     -5.9660310000    1.2608100000    0.5319360000
N      1.9083030000   -0.8778500000    1.9939240000
C      3.1816740000   -0.3609410000    1.7256570000
C      3.1885920000    1.0766730000    1.5081980000
C      3.9848060000   -0.3434260000   -1.7027370000
C      3.4394560000    0.8688010000   -1.9123800000
C      2.0182340000    1.0592630000   -1.6759720000
N      1.3200970000   -0.0963380000   -1.3394400000
C      1.8551670000   -1.3436070000   -1.1383150000
N      3.2176680000   -1.4223040000   -1.3507000000
C      2.0180720000    1.7435130000    1.4545250000
N      0.8144990000    1.1353750000    1.6743790000
C      0.6891030000   -0.2164990000    1.9536490000
O      4.1489880000   -1.1028050000    1.6610380000
O     -0.3725140000   -0.7688540000    2.1422030000
H      1.8447080000   -1.8813510000    2.1102140000
H      1.9558810000    2.7983520000    1.2138010000
H     -0.0388630000    1.6343970000    1.4206740000
H      4.1349960000    1.5652860000    1.3266800000
O      1.4418370000    2.1405010000   -1.7298820000
O      1.2041850000   -2.3186020000   -0.7909660000
H      0.3063600000    0.0121470000   -1.1167860000
H      4.0343470000    1.7262010000   -2.1931530000
H      3.6480340000   -2.2893520000   -1.0635900000
H      5.0456520000   -0.5399550000   -1.7979550000
```

```
41
DUU_pi_dimer_wB97XD_gs #BSSE corrected: Tab.S5
N     -1.0853630000   -2.2389190000    0.9754760000
N     -3.2657350000   -1.6274930000    0.5101120000
N     -5.3812570000   -0.6003790000   -0.1254720000
N     -4.7228540000    1.5288870000   -0.3661670000
N     -1.4929530000    0.0061620000    0.6205440000
N     -1.7982200000    2.2573130000    0.1421200000
C     -2.0083290000   -1.2394160000    0.7097820000
C     -4.0429420000   -0.5939610000    0.1670530000
C     -5.7246580000    0.6990800000   -0.4375000000
H     -6.7380490000    0.9625430000   -0.7081380000
C     -3.6593260000    0.7312990000    0.0152700000
C     -2.2986790000    1.0153320000    0.2509710000
H     -0.2984100000   -1.9530120000    1.5617700000
H     -1.5280570000   -3.0955200000    1.2808920000
H     -0.7907260000    2.3842760000    0.0651100000
H     -2.3944100000    2.9546830000   -0.2766230000
H     -5.9819440000   -1.4092590000   -0.1247940000
N      2.0289500000    0.0866840000   -2.2256370000
C      3.2838420000   -0.3040300000   -1.7410730000
C      3.2482180000   -1.5438190000   -0.9818180000
C      3.9351560000    0.9771330000    1.5729140000
C      3.3936090000   -0.1042880000    2.1623640000
C      1.9802130000   -0.3869300000    1.9720480000
N      1.2880600000    0.5732360000    1.2389230000
C      1.8140190000    1.6970960000    0.6516780000
N      3.1725030000    1.8575470000    0.8523340000
C      2.0621640000   -2.1305910000   -0.7261730000
N      0.8805510000   -1.6431480000   -1.2085930000
C      0.7949120000   -0.5029400000   -1.9916750000
O      4.2685160000    0.3848090000   -1.9495020000
O     -0.2460980000   -0.0625350000   -2.4263500000
H      1.9975140000    0.9520710000   -2.7492170000
H      1.9700780000   -3.0152440000   -0.1070520000
H      0.0075800000   -2.0110800000   -0.8313390000
H      4.1759880000   -1.9365940000   -0.5915660000
O      1.4035790000   -1.3860010000    2.3846560000
O      1.1600160000    2.4973700000    0.0021180000
H      0.2813040000    0.3869440000    1.0505310000
H      3.9873070000   -0.8034560000    2.7336850000
H      3.5981510000    2.6169450000    0.3418220000
H      4.9905980000    1.2145280000    1.6290250000
```

D:U□U (CPD)

```
41
DUU_dimer_wB97XD_gs #relevant for TD-DFT: Fig 5, Tab.S6
N     -0.8474840000    0.5943040000   -0.9377860000
C     -1.4535230000    1.8459220000   -0.8191250000
N     -2.7886600000    1.9123250000   -1.1094240000
H     -3.2006110000    2.7889460000   -0.8203820000
C     -3.6438110000    0.7652860000   -1.2005430000
H     -4.4759470000    0.9997290000   -1.8672120000
N      1.8190080000    2.5268130000    0.7613420000
N      3.8878500000    1.5384600000    0.7757190000
N      5.8742650000    0.1392790000    0.6418450000
N      5.0295440000   -1.6360890000   -0.4356920000
N      1.9764450000    0.4618370000   -0.2519020000
N      2.0992730000   -1.6327130000   -1.2544500000
C      2.6010860000    1.4762110000    0.4096450000
C      4.5533660000    0.4374070000    0.4245680000
C      6.0932540000   -1.1139590000    0.1046760000
H      7.0667610000   -1.5828260000    0.1498490000
C      4.0545760000   -0.6720530000   -0.2439860000
C      2.6886860000   -0.6255850000   -0.5874280000
H      0.9216140000    2.6633810000    0.3061510000
H      2.3050630000    3.3404440000    1.1017040000
H      1.0865110000   -1.6730330000   -1.3338700000
H      2.6316920000   -2.4788030000   -1.3826440000
H      6.5407730000    0.7286720000    1.1144710000
O     -0.8092290000    2.8315030000   -0.5063100000
C     -1.4434820000   -0.5654850000   -1.3421180000
O     -0.8283250000   -1.6127970000   -1.4732870000
C     -2.9284200000   -0.5341690000   -1.6098170000
H     -3.0835550000   -0.8752550000   -2.6354040000
H      0.1750420000    0.5630970000   -0.7127790000
C     -3.7674870000   -1.2942460000   -0.5202740000
H     -4.6171600000   -1.7924750000   -0.9914450000
C     -4.1756520000    0.0579340000    0.0972260000
H     -5.2395000000    0.1990410000    0.2984600000
N     -3.1407110000   -2.2136350000    0.3842100000
C     -3.4205730000    0.4600610000    1.3426590000
O     -3.5253490000    1.5699950000    1.8198770000
H     -2.8432730000   -3.1073500000    0.0180490000
C     -2.4151550000   -1.8335520000    1.4844700000
N     -2.5939790000   -0.4984700000    1.8830210000
O     -1.6948540000   -2.5769370000    2.1106670000
H     -2.0713900000   -0.2350840000    2.7095640000
```

41
DUU_dimer_wB97XD_gs #BSSE corrected: Tab.S5

N	-1.0853630000	-2.2389190000	0.9754760000
N	-3.2657350000	-1.6274930000	0.5101120000
N	-5.3812570000	-0.6003790000	-0.1254720000
N	-4.7228540000	1.5288870000	-0.3661670000
N	-1.4929530000	0.0061620000	0.6205440000
N	-1.7982200000	2.2573130000	0.1421200000
C	-2.0083290000	-1.2394160000	0.7097820000
C	-4.0429420000	-0.5939610000	0.1670530000
C	-5.7246580000	0.6990800000	-0.4375000000
H	-6.7380490000	0.9625430000	-0.7081380000
C	-3.6593260000	0.7312990000	0.0152700000
C	-2.2986790000	1.0153320000	0.2509710000
H	-0.2984100000	-1.9530120000	1.5617700000
H	-1.5280570000	-3.0955200000	1.2808920000
H	-0.7907260000	2.3842760000	0.0651100000
H	-2.3944100000	2.9546830000	-0.2766230000
H	-5.9819440000	-1.4092590000	-0.1247940000
N	2.0289500000	0.0866840000	-2.2256370000
C	3.2838420000	-0.3040300000	-1.7410730000
C	3.2482180000	-1.5438190000	-0.9818180000
C	3.9351560000	0.9771330000	1.5729140000
C	3.3936090000	-0.1042880000	2.1623640000
C	1.9802130000	-0.3869300000	1.9720480000
N	1.2880600000	0.5732360000	1.2389230000
C	1.8140190000	1.6970960000	0.6516780000
N	3.1725030000	1.8575470000	0.8523340000
C	2.0621640000	-2.1305910000	-0.7261730000
N	0.8805510000	-1.6431480000	-1.2085930000
C	0.7949120000	-0.5029400000	-1.9916750000
O	4.2685160000	0.3848090000	-1.9495020000
O	-0.2460980000	-0.0625350000	-2.4263500000
H	1.9975140000	0.9520710000	-2.7492170000
H	1.9700780000	-3.0152440000	-0.1070520000
H	0.0075800000	-2.0110800000	-0.8313390000
H	4.1759880000	-1.9365940000	-0.5915660000
O	1.4035790000	-1.3860010000	2.3846560000
O	1.1600160000	2.4973700000	0.0021180000
H	0.2813040000	0.3869440000	1.0505310000
H	3.9873070000	-0.8034560000	2.7336850000
H	3.5981510000	2.6169450000	0.3418220000
H	4.9905980000	1.2145280000	1.6290250000

UU CI

```
24
U_U_CASSCF_optconical
N      1.9180330000   -0.0416640000   -0.8163020000
C      2.0396900000    0.7287140000    0.3329150000
C      1.3580810000    0.1934200000    1.4996910000
C     -0.6867550000    1.0807270000    1.4061520000
C     -1.3571850000   -0.1943180000    1.5000950000
C     -2.0393880000   -0.7289780000    0.3335210000
N     -1.9184570000    0.0421450000   -0.8153750000
C     -1.3795750000    1.3009080000   -0.9228130000
N     -0.8267600000    1.7942050000    0.2394260000
C      0.6882530000   -1.0818680000    1.4053830000
N      0.8276770000   -1.7947430000    0.2382680000
C      1.3785570000   -1.3001610000   -0.9243810000
O      2.6212570000    1.7744290000    0.3018790000
O      1.3851980000   -1.9248940000   -1.9376020000
H      2.3435850000    0.3165550000   -1.6456480000
H      0.5231260000   -1.6771810000    2.2795580000
H      0.3597940000   -2.6693530000    0.1313070000
H      1.6926140000    0.5434230000    2.4560240000
O     -2.6208870000   -1.7747300000    0.3021100000
O     -1.3875600000    1.9266910000   -1.9354130000
H     -2.3449920000   -0.3153540000   -1.6445230000
H     -1.6904430000   -0.5452750000    2.4565230000
H     -0.3597360000    2.6693100000    0.1328730000
H     -0.5215180000    1.6756310000    2.2805960000
```

D:UU CI

```
41
DU_U_CASSCF_optconical
N     -0.7232240000   -0.3994030000   -0.9139490000
C     -1.4257110000    0.6156780000   -1.4999030000
N     -2.7153690000    0.3201360000   -1.8579410000
H     -3.2316140000    1.0959880000   -2.2129530000
C     -3.3824230000   -0.8614030000   -1.5351350000
H     -4.1928090000   -1.0778810000   -2.2063400000
N      1.8842870000    2.4136390000   -0.6556390000
N      4.0203340000    1.7937720000   -0.1867550000
N      6.1312050000    0.7634820000    0.3773700000
N      5.5021320000   -1.3563540000    0.5494230000
N      2.2522890000    0.1824140000   -0.3138970000
N      2.5786110000   -2.0676280000    0.0567440000
C      2.7616390000    1.4275090000   -0.3803010000
C      4.7973730000    0.7631960000    0.1127800000
C      6.4833430000   -0.5432590000    0.6310580000
H      7.4928820000   -0.8091960000    0.8695960000
C      4.4209630000   -0.5539840000    0.2219930000
C      3.0643710000   -0.8280470000   -0.0125300000
H      0.9795930000    2.1959530000   -1.0133810000
H      2.2670450000    3.3064880000   -0.8621000000
H      1.6073710000   -2.2571900000   -0.0840140000
H      3.1921230000   -2.8125020000    0.2930620000
H      6.7241930000    1.5610260000    0.3880810000
O     -0.9594700000    1.7034660000   -1.6954170000
C     -1.1486870000   -1.7109050000   -0.7791620000
O     -0.3889250000   -2.5558240000   -0.3693980000
C     -2.5268940000   -1.9558610000   -1.1211680000
H     -2.8497820000   -2.9719870000   -1.2203250000
H      0.2445580000   -0.1983770000   -0.6919560000
C     -3.7183080000   -1.3988300000    0.9959970000
H     -3.8999310000   -2.4443930000    1.1230380000
C     -4.5055990000   -0.5776370000    0.1064990000
H     -5.4580560000   -0.9732100000   -0.1974410000
N     -2.9207350000   -0.8091460000    1.9183080000
C     -4.5160170000    0.8729390000    0.3527260000
O     -5.2015660000    1.6459010000   -0.2468640000
H     -2.3197670000   -1.3583990000    2.4957440000
C     -2.7979180000    0.5583550000    2.1046480000
N     -3.5978450000    1.3187830000    1.2885430000
O     -2.0677550000    1.0155230000    2.9211260000
H     -3.5326570000    2.3067680000    1.4229460000
```

UU CI

```
24
input_qU_U_structure_from_scan_2.1_2.4
N      1.7477580000    0.6193640000   -0.7841990000
C      1.3948130000    1.3997560000    0.3045340000
C      0.9884780000    0.6647110000    1.5095790000
C     -1.1110750000    0.6549990000    1.4673570000
C     -1.2896090000   -0.8443560000    1.3919140000
C     -1.6040670000   -1.4401090000    0.0890900000
N     -1.6826620000   -0.5365660000   -0.9594410000
C     -1.7968760000    0.8321550000   -0.8684700000
N     -1.6465160000    1.3524280000    0.4022220000
C      1.1090000000   -0.8393770000    1.4734840000
N      1.7946420000   -1.3784360000    0.3993600000
C      2.0239510000   -0.7285790000   -0.7892260000
O      1.3555790000    2.5915350000    0.2074960000
O      2.4517060000   -1.2898320000   -1.7523140000
H      1.9663670000    1.1020130000   -1.6307650000
H      1.2670360000   -1.3605120000    2.3972830000
H      1.9363970000   -2.3641740000    0.3491060000
H      1.3317070000    1.1424430000    2.4123330000
O     -1.7491950000   -2.6094550000   -0.0973220000
O     -2.0337440000    1.5195300000   -1.8095100000
H     -1.8612830000   -0.9178570000   -1.8651550000
H     -1.6884760000   -1.3552280000    2.2469580000
H     -1.6108730000    2.3502130000    0.4146420000
H     -1.3158830000    1.1161220000    2.4136290000
```

D:UU starting guess structure for CI opt

```
41
DU_U_structure_from_scan_2.1_2.1
N     -0.8676160000    0.5095860000   -1.0225630000
C     -1.4594330000    1.7528880000   -0.9953880000
N     -2.7647810000    1.8101660000   -1.4018970000
H     -3.1845140000    2.7017330000   -1.2490380000
C     -3.5910510000    0.6911550000   -1.5580800000
H     -4.4466760000    0.8981180000   -2.1740140000
N      1.8276600000    2.3881830000    0.9312680000
N      3.9287310000    1.4782850000    0.9261900000
N      5.9703810000    0.1763750000    0.7589380000
N      5.2432030000   -1.5234200000   -0.4629770000
N      2.1138010000    0.4192890000   -0.2287900000
N      2.3401740000   -1.5668830000   -1.3948930000
C      2.6752320000    1.3994550000    0.5229710000
C      4.6546570000    0.4329330000    0.5197790000
C      6.2511040000   -1.0144540000    0.1396900000
H      7.2309690000   -1.4445470000    0.1795840000
C      4.2202810000   -0.6210330000   -0.2358340000
C      2.8663740000   -0.5982490000   -0.6211440000
H      1.0070470000    2.5507750000    0.3876660000
H      2.2765400000    3.2037340000    1.2819650000
H      1.3479540000   -1.6531490000   -1.4649850000
H      2.8797990000   -2.3926420000   -1.5218330000
H      6.5925460000    0.7423370000    1.2886380000
O     -0.8574720000    2.7306380000   -0.6597620000
C     -1.4141760000   -0.6471180000   -1.5142970000
O     -0.7638830000   -1.6475450000   -1.6318110000
C     -2.8576690000   -0.6035830000   -1.8494650000
H     -3.0746840000   -1.0961730000   -2.7820370000
H      0.1093950000    0.4854060000   -0.7547580000
C     -3.8980970000   -1.5054400000   -0.2638540000
H     -4.5945730000   -2.1453480000   -0.7734530000
C     -4.4357770000   -0.1538150000    0.1689000000
H     -5.5054530000   -0.0299680000    0.1861930000
N     -3.1670140000   -2.1901060000    0.7124670000
C     -3.7750670000    0.4914390000    1.3304850000
O     -4.0462590000    1.5907520000    1.7054920000
H     -2.8433630000   -3.1124860000    0.5159520000
C     -2.4804320000   -1.5831840000    1.7325380000
N     -2.7807850000   -0.2485290000    1.9335980000
O     -1.6994180000   -2.1620300000    2.4192940000
H     -2.3006930000    0.1810970000    2.6972280000
```

References

(1) Dhaouadi, Z.; Ghomi, M.; Mojzes, P.; Turpin, P. Y.; Chinsky, L. Vibrational mode analysis of 2-aminoadenine and its deuterated species from Raman and ultraviolet resonance Raman data. *Eur. Biophys. J.* **1994**, *23*, 95–104.

(2) Yarasi, S.; Ng, S.; Loppnow, G. R. Initial Excited-State Structural Dynamics of Uracil from Resonance Raman Spectroscopy Are Different from Those of Thymine (5-Methyluracil). *J. Phys. Chem. B* **2009**, *113*, 14336–14342.

(3) Simon, S.; Duran, M.; Dannenberg, J. J. How does basis set superposition error change the potential surfaces for hydrogen-bonded dimers? *The Journal of Chemical Physics* **1996**, *105*, 11024–11031.
